# The effects of Antibody Engineering CH and CL in Trastuzumab and Pertuzumab recombinant models: Impact on antibody production and antigen-binding

**DOI:** 10.1038/s41598-017-18892-9

**Published:** 2018-01-15

**Authors:** Wai-Heng Lua, Wei-Li Ling, Joshua Yi Yeo, Jun-Jie Poh, David Philip Lane, Samuel Ken-En Gan

**Affiliations:** 1 Bioinformatics Institute, Agency for Science, Technology and Research (A*STAR), Singapore, Singapore; 2 p53 Laboratory, Agency for Science, Technology and Research (A*STAR), Singapore, Singapore

## Abstract

Current therapeutic antibodies such as Trastuzumab, are typically of the blood circulatory IgG1 class (Cκ/ CHγ1). Due to the binding to Her2 also present on normal cell surfaces, side effects such as cardiac failure can sometimes be associated with such targeted therapy. Using antibody isotype swapping, it may be possible to reduce systemic circulation through increased tissue localization, thereby minimising unwanted side effects. However, the effects of such modifications have yet to be fully characterized, particularly with regards to their biophysical properties in antigen binding. To do this, we produced all light and heavy chain human isotypes/subtypes recombinant versions of Trastuzumab and Pertuzumab, and studied them with respect to recombinant production and Her2 binding. Our findings show that while the light chain constant region changes have no major effects on production or Her2 binding, some heavy chain isotypes, in particularly, IgM and IgD isotypes, can modulate antigen binding. This study thus provides the groundwork for such isotype modifications to be performed in the future to yield therapeutics of higher efficacy and efficiency.

## Introduction

The “new dawn”^1^ of therapeutics had come with recombinant monoclonal antibodies (mAbs). Most approved clinical therapeutic mAbs are of the Cκ and Cγ1 isotypes, notably Trastuzumab and Pertuzumab which have significant combined success in the treatment of Her2 + cancers^2^. However, when bound to normal Her2 expressing cardiac cells, Trastuzumab can cause side effects such as cardiac failure^3^. To reduce such side effects, one possible solution is to improve the antibody localization to the cancer target areas, reducing systemic circulation. Such efforts can be actualized by engineering a change of the antibody isotype through recombinant means, especially since the general localization of these antibody isotypes are already well established in classic immunology. On this possibility, there is great interest in utilizing isotypes for immunotherapy, particularly for cancer^4^.

The human immunoglobulin (Ig) heavy chain isotypes and subtypes (CH variants) consist of IgM, IgA1, IgA2, IgD, IgG1, IgG2, IgG3, IgG4, and IgE. Of the CH variants, the most abundant is the IgG isotype in which IgG1 is the most dominant subtype in blood. IgM, like IgG, is also predominantly localized in blood and both isotypes exhibit specialized immune functions such as Antibody Dependent Cell-mediated Cytotoxicity (ADCC)^5^. Given its role in neutralizing infectious agents^5^ and activating the complement system amongst the recruitment of immune cells, IgG, particularly IgG1, is the default choice for therapeutic monoclonal antibodies.

Nonetheless, there has been increasing interest in exploring the use of alternative CH variants as therapeutic antibodies. Two such examples include IgA^6^ and IgE^7^, along with their immune activation mechanisms^8,9^. With the prowess of these CH variants to also elicit immune responses at a level comparable to IgG, there are potential advantages in using these CH variants when considering their localization in tissues or organs of interest.

IgM, the primary antibody responsible for defense against new antigens, is often found as an oligomer (pentamer or hexamer) with or without the J-chain. It functions to agglutinate and immobilize antigens^10^ as well as activate the complement system^11^. IgA (both A1 and A2), the principal CH variants found in mucosal areas (intestinal, respiratory tracts, lactating breast and various exocrine glands such as tear and salivary glands), exists in monomeric or dimeric forms and plays the key role in adaptive mucosal immunity^5,12^. IgD is the least characterized CH variant, and exists in two forms: membrane bound and secreted. While the membrane bound form typically serves as the B cell receptor^13,14^, the secreted form is found in the upper respiratory tract^13,14^, and to a less extent, in the bone marrow^13^. The least abundant CH variant, IgE, is responsible for allergies such as asthma via mast cell activation. It is also found in mucosal areas such as gastrointestinal, respiratory tracts, and on skin to mediate the allergic response. Possibilities to utilize IgE to make patients allergic to their cancer in “AllergoOncology”^15^ have recently made IgE a popular potential therapeutic target.

While the specific localization and immune functions are generally specified by the CH variants or Fc region of the antibodies^15^, much remains unexplored to what the effects of antibody class-switching can have on recombinant production and antigen binding. According to a previous study^16^ on antigen binding, the researchers found minimal effect on antigen-binding kinetics for IgGs, but there has yet to be a comprehensive biophysical characterization of all known human CH variants.

To examine the effects of the light chain, particularly the light chain constant region (CL), we produced Trastuzumab and Pertuzumab recombinant antibodies with the κ constant (Cκ), and five functional λ constant (Cλ) regions^17^. Compared to the CH variety, the human CL has no clear immunological or localization roles. Nonetheless, a recent study did suggest that CL can structurally influence the antibody elbow region, and thus possibly antigen binding^18^. Given that the light chain of Trastuzumab have been found to mediate antibody dissociation^19^ from Her2 where VL can affect dissociation rates (Kd), the different Cλs may also display such effects given the findings of another study showing that swapping light chain elements can affect structural stability^20^.

To explore the potential of isotype swapping in antibody engineering for future therapeutics, we investigated the effect of the CH CL regions in recombinant antibody production and antigen binding. In order to do this, we used Trastuzumab and Pertuzumab as recombinant models, grafting their light (VL) and heavy (VH) variable regions onto the known and validated human CHs and CLs for the biophysical characterizations.

## Results

We made the nine recombinant CH and six CL (one Cκ, and 5 Cλ) isotype/subtype variants of two well-studied clinical monoclonal antibodies: Trastuzumab and Pertuzumab. Our initial attempts to produce the CH variants in *E.coli* as previously reported^21^ for IgG1 using pET21(d) did not yield detectable Ig production (not shown) and thus we performed transient co-transfection into HEK293F cells instead. Protein G (for IgG and Cλ subtypes) and Protein L (for the non-IgG types through capture of the κ-light chain) columns were used in affinity chromatography followed by size exclusion chromatography. CH variant comparisons were performed only with the original Cκ isotype.

### CH isotype effects

Recombinant production analysis of the nine CH variants combined with the original Cκ light chain showed that the majority of the CH variants antibodies were secreted by the transfected cells in decent quantities when compared to the original IgG1 counterparts. A general trend of production rate for both Trastuzumab/Pertuzumab is as follows: IgAs > IgGs > IgM > IgE > IgD. IgE and IgD recombinant productions were lower compared to those of IgA and IgG (~20 times lower) for both antibody models. We found both Trastuzumab and Pertuzumab IgD variants to consistently give the lowest yields among all CH variants produced. Higher proportions of oligomerization were observed for IgD (due to the general lower yield for IgD) when compared to the IgA and IgE CH variants, with the expected exception of IgM.

**Figure 1:**
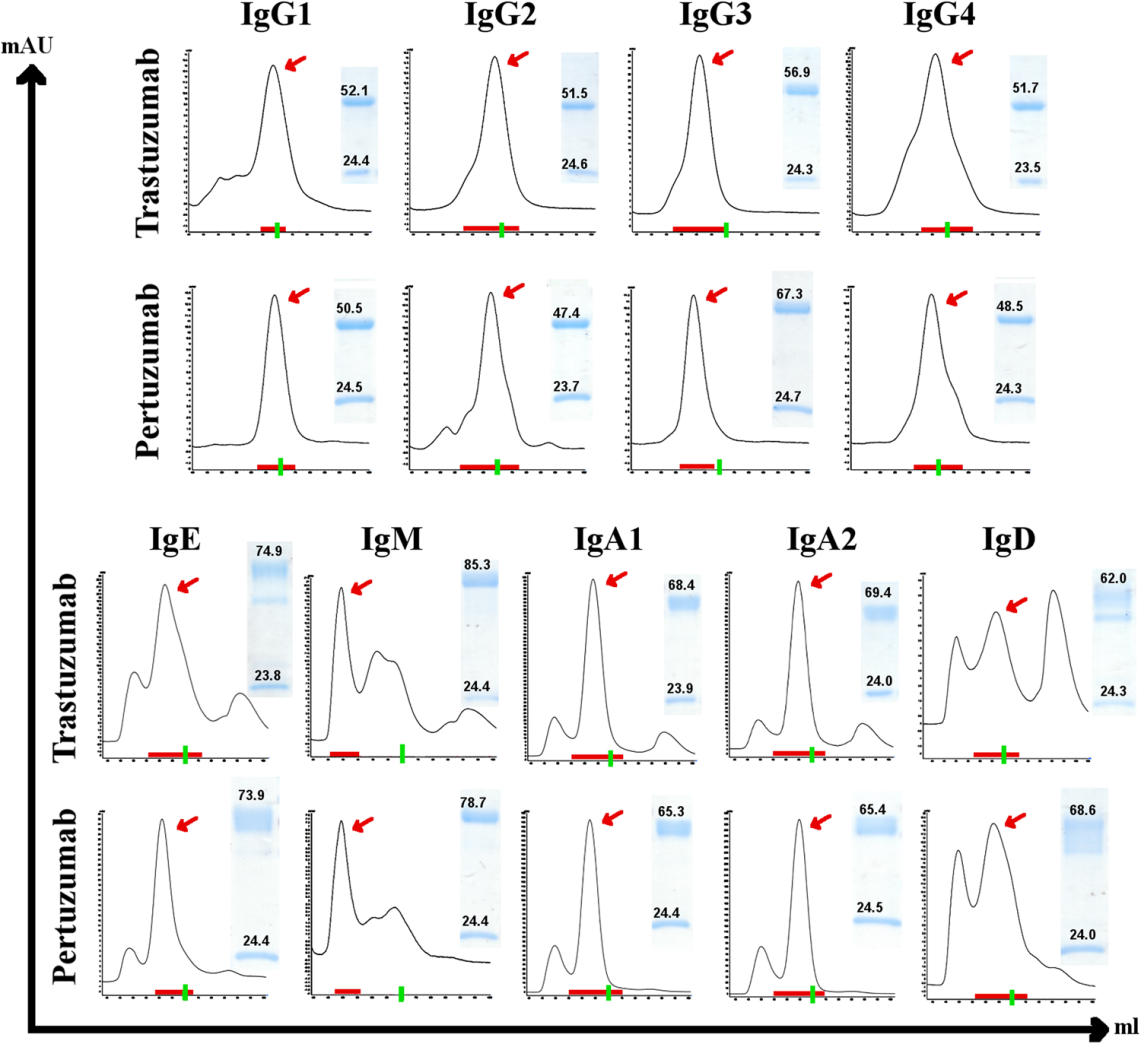
Trastuzumab and Pertuzumab isotype/subtype characterization. Size Exclusion Chromatography profiles of the Trastuzumab and Pertuzumab variants following affinity purification using Superdex size exclusion column on the AKTA Pure system. X-Axis: 40–100 ml time scale. Y-axis: mAU absorption as determined by UV detection. The green lines on the X-axis marks the 70 ml mark which corresponds to ~150 kDa as determined by previous calibrations (see Supplementary Data). Red arrows depict the selected peak fractions of the antibodies used for subsequent analyses while the red horizontal line at the x-axis indicate the fractions collected. SDS-PAGE analyses of concentrated antibodies (reduced) are quantified and shown in the smaller inserts with corresponding band sizes for the light and heavy chain (for gel photos with ladder analysed with GelApp2.0, see Supplementary Data) using GelApp2.0. Graphs are representative of at least 3 independent batches of transfected cells.

With the exception of IgM, and to a certain extent, IgD, all other CH variants were produced predominantly as monomers (determined by elution volume on the size exclusion chromatography, as shown in Supplementary Data) with low levels of aggregation/oligomerization. The high oligomerization of IgM (Fig. 1) is expected given the isotype penta/hexameric nature^10^, and this was also in agreement to the expected elution size (for penta/hexamers at ~150 kDa x 5 or 6, see Supplementary Data).

**Figure 2:**
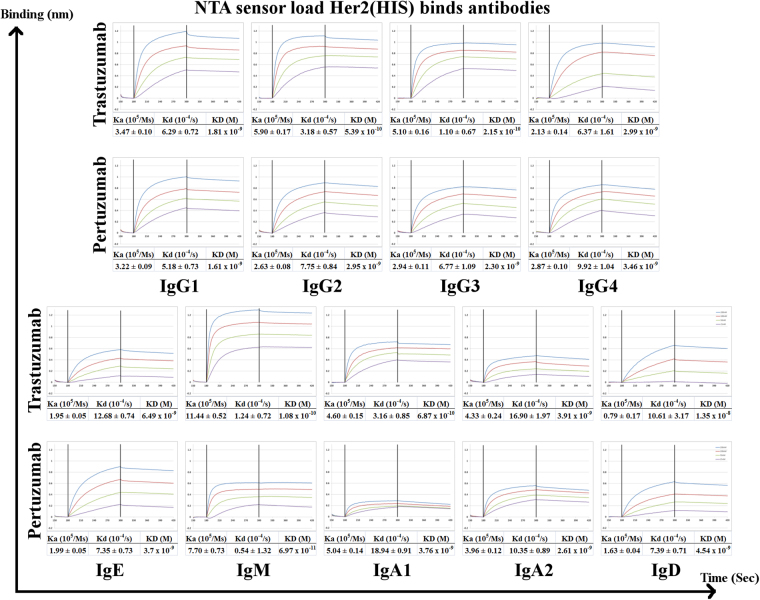
Binding affinity measurements of Trastuzumab and Pertuzumab isotype/subtype variants to Her2, using antibodies at 200 nM to 25 nM to pre-loaded Her2 on NTA biosensors. KD, Ka, and Kd were measured and calculated by the the Blitz® software. All experiments were performed with at least three independent occasions in triplicate readings. The X-axis depicts the time in seconds. The Y-axis depicts the binding in nm.

**Figure 3:**
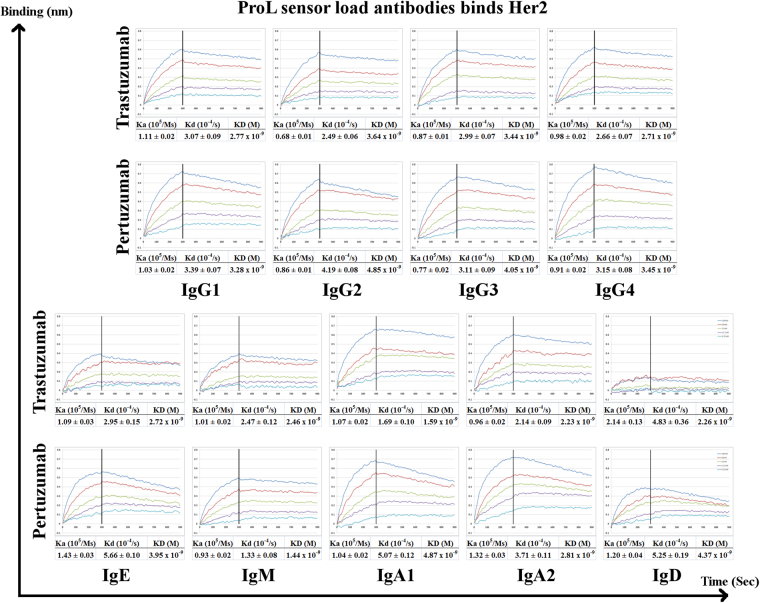
Binding affinity measurements of Trastuzumab and Pertuzumab isotype/subtype variants to Her2 at 100 nM to 6.25 nM concentrations to pre-loaded Trastuzumab and Pertuzumab variants on Protein L (ProL) biosensors. KD, Ka, and Kd were measured and calculated by the Octet QK^e^ system. All experiments were performed with at least three independent occasions in triplicate readings. The X-axis depicts the time in seconds. The Y-axis depicts the binding in nm.

Analyses of binding kinetics of the Trastuzumab and Pertuzumab CH variants were conducted by pre-loading Her2 to the sensor followed by the binding of the antibodies (Fig. 2) or by pre-loading the antibodies to the sensors followed by binding to Her2 (Fig. 3). Both methods showed that CH variants, with the exception of IgD and IgM, generally exhibited similar equilibrium dissociation constants (KD, calculated by kd/ka) to the original IgG1 Trastuzumab and Pertuzumab counterparts (Figs 2 and 3). Within the KD values of the Trastuzumab IgG subtype binding to pre-loaded Her2 biosensors (Fig. 2), IgG2 and IgG3 subtypes were 1-log smaller than the IgG1 counterpart (Fig. 2), although this difference was not observed with the Protein L pre-loaded IgG antibodies binding to Her2. The findings regarding the IgG variants agree with previous studies of the respective antibodies^22–24^ where there is generally little difference between the IgG subtypes. Due to avidity effects (demonstrated by the differences when measuring the same antibody when Her2 was preloaded or when the antibody was pre-loaded by Protein L), only Pertuzumab IgM showed more than 2-log difference in the equilibrium dissociation constant (KD) to the IgG counterpart when Her2 was preloaded to the sensor. This was the only exception to the general observation where Trastuzumab variants exhibited higher affinities to Her2 than the Pertuzumab variants, which was previously determined for the commercial counterparts^25^.

When comparing within the pre-loaded Her2 set (Fig. 2), Trastuzumab IgD had 1–2 log difference when compared to the equilibrium dissociation constants of the IgG isotype. This was not observed for the Pertuzmab IgD. This discrepancy suggests that while the isotype may have effects on the binding ability of the antibody, the Complementarily Determining Regions (CDR) of the antibodies can mitigate this effect. This is especially important since both Trastuzumab and Pertuzumab utilize VH3 and Vκ1 families. This difference was lost when the experiment set up was reversed to having pre-loaded antibodies to the Protein L biosensor (Fig. 3), suggesting that avidity effects were at play.

We found very little difference in the association rates (Ka or Kon) between the isotypes for Pertuzumab when comparing the general IgG subtype with that to the others regardless of the experiment setup. Trastuzumab isotype variants appeared to be more prone to avidity effects given that IgM and IgD had more fluctuations in the association constant when the experiment setup reversed. Specifically, Trastuzumab IgD binding to protein L seemed to be affected by the isotype change, with the IgD dropping off when compared to the other isotypes. Therefore the measurements for Trastuzumab IgD assay on protein L is less reliable than then that measured by its binding to pre-loaded Her2.

Variability in the dissociation rate (Kd or Koff) between the CH variants were more pronounced on both Trastuzumab and Pertuzumab. Trastuzumab IgE, IgA2 and IgD exhibited 1-log difference when bound to pre-loaded Her2 but not when the antibody was pre-loaded, again alluding to avidity effects. For Pertuzumab, this was observed for the IgM, IgA1 and IgA2.

### CL comparison

**Figure 4:**
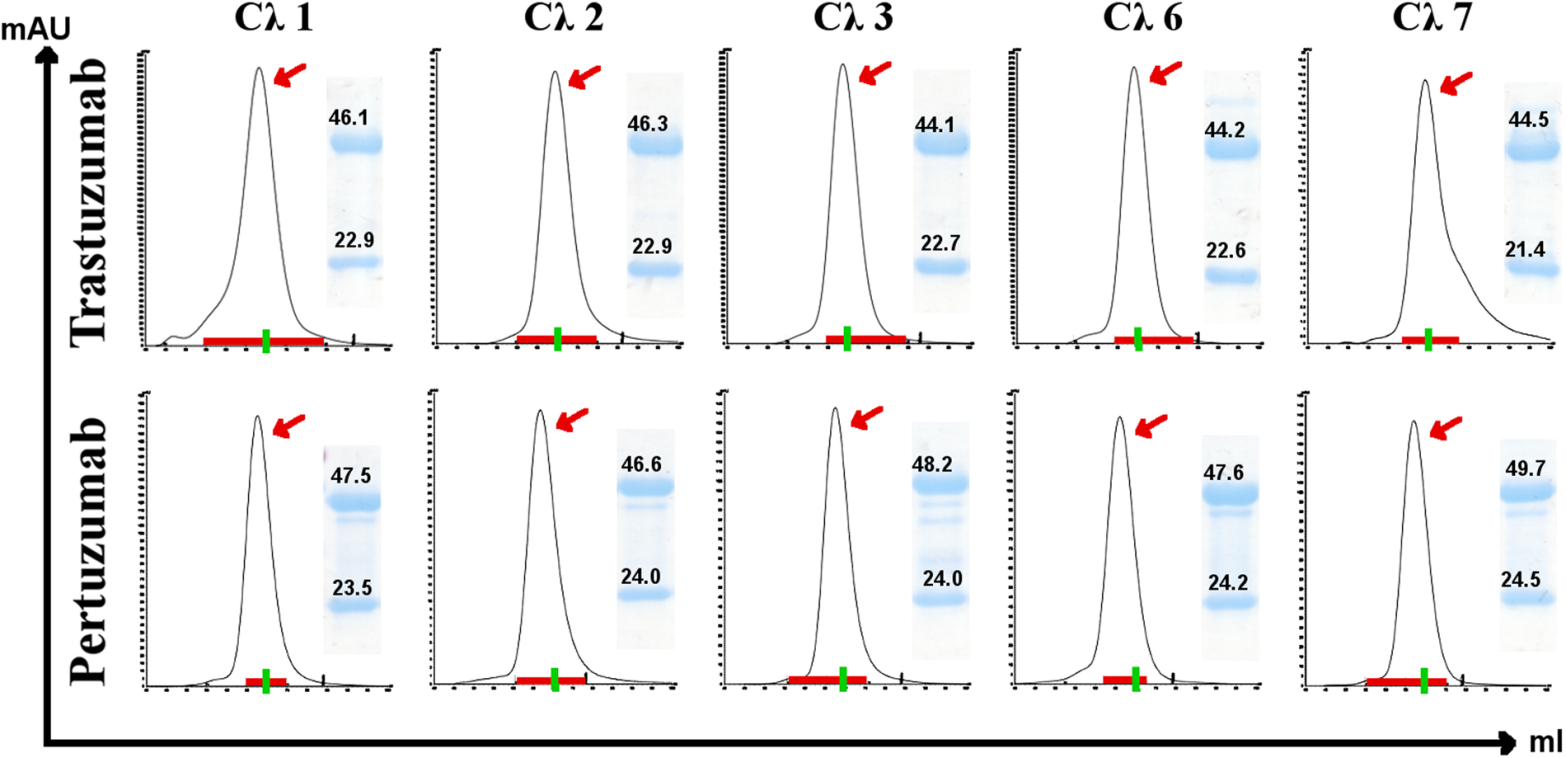
Trastuzumab and Pertuzumab Cλ light chain isotype characterization. Size Exclusion Chromatography profiles of the Trastuzumab and Pertuzumab variants following affinity purification using Superdex size exclusion column on AKTA Pure. X-Axis: 40–100 ml scale. Y-axis: mAU aborption as determined by UV detection. The green lines on the X-axis marks the 70 ml mark which corresponds to ~150 kDa as determined by previous calibrations (see Supplementary Data). Red arrows depict the selected peak fractions of the antibodies used for subsequent analyses while the red horizontal line at the x-axis indicates the fractions collected. SDS-PAGE analyses of concentrated antibodies (reduced) are quantified and shown in the smaller inserts with corresponding band sizes for the light and heavy chains (for gel photos with ladder analysed with GelApp2.0, see Supplementary Data) using GelApp2.0. Graphs are representative of at least 3 independent batches of transfected cells.

The Trastuzumab and Pertuzumab VκCλ variants of CL were produced retaining the original Vκ1. Despite having chimeric light-chains, all the five IgG1-paired Cλ families were purified predominantly in monomeric forms (as determined by the elution on a pre-calibrated column, Fig. 4 and Supplementary Data) when compared to the Cκ original. The production rates of the CL variants from the transfected cells were similar to the original Cκ with the exception of the CLλ7 variant that was produced at lower levels (approximately 2-folds lower given the varying rates in transient transfections).

**Figure 5:**
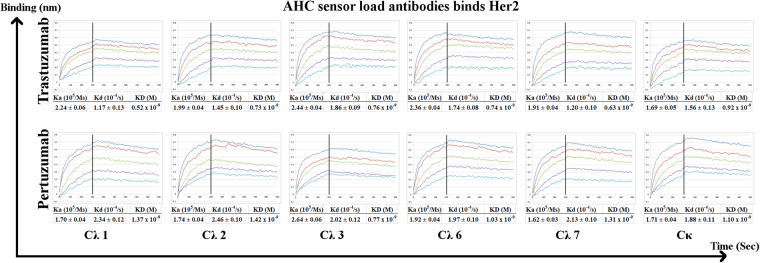
Binding affinity measurements of Trastuzumab and Pertuzumab λ light chain isotype variants to Her2 at 100 nM to 6.25 nM concentrations to Trastuzumab or Pertuzumab variants pre-loaded on AHC biosensors. KD, Ka, and Kd were measured and calculated by the Octet QK^e^ system. All experiments were performed with at least three independent occasions in triplicate readings., graphs shown were representative of each variant. The X-axis depicts the time in seconds. The Y-axis depicts the binding in nm.

The equilibrium dissociation constant (KD), association rate (Ka), and dissociation rate (Kd) of the CL variants did not show significant differences between the Cλ chimera variants when compared to the original Cκ. The Trastuzumab Cλ variants had a KD of 0.52 to 0.92 × 10^−9^ (M) while the Pertuzumab Cλ variants had a KD of 0.77 to 1.42 × 10^−9^ (M) (Fig. 5). Both Trastuzumab and Pertuzumab chimeric Cλ variants readings were comparable to the respective original Cκ variant as well as previously measured clinical therapeutics^25^.

## Discussion

We set out to biophysically characterize all human six light and nine heavy chain isotypes and subtypes with regards to recombinant production and antigen-binding kinetics. Our attempts with bacterial expression of these isotypes did not result in detectable protein productions, even when applied for whole IgG1 as previously reported^21^. Given the human nature of the antibodies requiring post-translational modification (a factor we wanted to rule out), the failure of bacterial expression was inconsequential. The reasons for the failed bacterial expression may be attributed to the different bacterial plasmid used, as well as the different variable sequences.

To our knowledge, our study is one of the first comprehensive comparison of all known human heavy and light chain isotypes and subtypes utilizing two model antibodies against the same antigen simultaneously. It should be noted that the numbering of the Cλ families do not run in order because some Cλ families (e.g. Cλ4 & Cλ5) were later found to be pseudogenes or theoretical genes^17^. In fact, to solely investigate the effects of the CL, we kept the original VL, forming Vκ– Cλ hybrid variants.

On the heavy chain isotypes, IgM expectedly displayed distinct oligomerization to predominantly pemtameric/hexameric forms with some intermediates^10^ even without co-transfection of J-chain plasmids (Fig. 1). Such oligomerization for IgM is immunologically superior in activating the complement system than monomeric forms^11^, making IgM variants suitable candidates for targeting circulatory metastatic cancer cells that would be abated by immobilization of antigens via agglutination. Although IgG4 was previously reported to exhibit Fab arms exchange *in vivo*^16,26^, such exchange would not have an effect in our monoclonal expression methods, maintaining the validity of our biophysical characterizations.

The comparison of the association and dissociation rates of the CH variants to their commercial counterparts (Figs 2 and 3) shows the general feasibility of CH isotype switching for both Trastuzumab and Pertuzumab, and possibly for all therapeutic antibodies. Given that the immune functions of secreted IgD remain enigmatic and the poor protein production profiles of the Trastuzumab and Pertuzumab IgD variants, there may be need to reconsider the use of secreted IgD as a therapeutic variant. The fact that murine IgD receptor does not bind to human IgD^27^ while the human IgD receptor can bind both human IgD and IgA1 with the similar O-glycans modification at the hinge region^28^, makes IgD difficult to study using animal models. To remove the confounding factor of glycosylation, we produced all our CH variants in human HEK cell-lines over animal CHO or COS cells.

Interestingly, the avidity effects of Pertuzumab IgM reversed the general trend of poorer binding over Trastuzumab. While this only happened for the Pertuzumab model, and thereby we are cautious to generalize this to all antibodies, such observations do advocate for the use of IgM as a potential CH variant when working with weaker binding antibody candidates. Although IgG has been the predominant choice for therapeutic antibodies, IgM may yet be more suitable than IgG when it can increase overall binding strength and its superior agglutination effects when it comes to circulating antigens. However, the use of IgM as a therapeutics drug included a historical setback^29^, and the lack of a suitable animal model further present as major obstacles. While there are multiple receptors for IgM such as Fcα/μR and FcμR, and that human Fcα/μR binds both human and mouse IgM and IgA, it is expressed on different cell types compared to mice. In human, only pre-germinal centre B-cells (IgD + /CD38 + cells) express the receptors, while the murine counterparts are only expressed on circulating and resident B cell population^30^. Cross reactivity of the murine Fcα/μR to human IgM and IgA is not yet established, rendering this receptor unsuitable for animal model investigations. Nonetheless, a recently discovered receptor - FcμR(TOSO/FAIM3)^31,32^ - present on both human and mice, have demonstrated cross-species IgM reactivity. There are however also differences in the receptor localisation. It is expressed in both human and murine spleen and thymus, but also on peripheral blood leukocytes for humans, and bone marrow and lymph nodes for mice^31,32^.Given such differences, it is therefore unlikely that the mouse model would be suitable for IgM investigations, especially for localization experiments.

Our other CH variants, IgA1, IgA2, and IgE showed similar dissociation equilibrium constants, association and dissociation rate readings to the IgG1 subtype when pre-loaded to Protein L sensors, making them good possible candidates for mucosal cancers and AllergoOncology (IgE). There are hints of avidity effects when we compared the results to preloaded antigen (Her2), Trastuzumab isotype variants displayed more avidity effects where the IgM and IgD variants had more fluctuations in the association constant, but not for the Pertuzumab isotype variants. Similarly, variability in the dissociation rates between the CH variants, being more pronounced on both antibody models, had differences where for Trastuzumab, IgE, IgA2 and IgD showed 1-log difference when bound to pre-loaded Her2, whereas for Pertuzumab, the differences in the Kd were more pronounced for IgM, IgA1 and IgA2.

Other than to narrow it down to isotype-CDR-linked effects, we were unable to determine the cause of these avidity differences. Nonetheless, the pre-loading of the antibody variants on the Protein L have shown that the V-regions of these isotype variants were functional in order to be recognized by Protein L and also bind Her2.

Since many Her2 + cancers occur in ductal areas, and our results did not exclude the suitability of IgE and IgA isotype variants, the availability of such Trastuzumab/Pertuzumab IgE and IgA variants may be ideal for such localities. These variants can be used in conjunction with reduced dosage of blood-based IgM/IgG variants to check circulating cancer metastasis, and in the process, reduce cardiac side effects associated with high doses of Trastuzumab IgG1 alone. However, the lack of a mouse Ig receptor that responds to human IgA^12^ and IgE^15^ are perhaps the main reasons to why therapeutic antibodies of such isotypes are not yet common, and may not be approved for clinical trials in the near future.

Although the results of the IgG variants are similar, various human IgG subtypes (IgG2 and IgG4) exhibited different protection properties in mice against *Crytococcus neoformans*^22^ (encapsulated yeast). Since all human IgG subtypes can cross placenta^33^, therapeutics of this isotype is not likely to be suitable for use during pregnancy, thus advocating for the use of other isotypes to reduce possibilities of affecting the unborn and thus the withholding of targeted therapies during pregnancies.

As proof of concept in different localization, our preliminary experiments (not shown) in nude mice showed that IgG stayed longer in systemic circulation compared with the other isotypes, which were cleared in the kidneys and livers within the first few days. However this result is inconclusive as localization experiments in mice are severely limited given that mice Ig receptors for the other isotypes do not interact with the human Ig variants.

Nevertheless, the comparison of the different CH variants showed a trend of general better binding measurements when using the human IgG Fc capture AHC biosensors (Fig. 5) over that of Protein L biosensors (Fig. 3). Since our previous work on Trastuzumab light chains^19^ showed that VL frameworks can affect protein L binding, we sought to rule out interferences elicited by Protein L to the Her2 binding site by using AHC instead of Protein L when focusing on light chain analysis (Fig. 3).

It remains enigmatic to why the human Ig system has 5 families of Cλ but only one Cκ. Although studies have shown that free floating light chains are associated with inflammation responses and autoimmunity^34^, the role of the light chain has been fairly elusive with the lack of known specific Igκ and Igλ receptors,. Light chains have been found to affect the *in vivo* half-life of antibodies (where huIgG2λ had a shorter half-life than huIgG2κ in mice)^35^, as well as play a role in antigen dissociation^19^. For this reason, it may be that the effects of CL on antibody half-life and antigen-binding is not solely dependent only on CL-CH, but must include the other regions as whole heavy and light chains. This demonstrates the need for detailed analysis of how the variable and constant regions of the antibodies of both chains come together to affect antigen-binding, production and also half-lives.

Our CL findings are consistent with Montaño *et al*.^35^ (where the CL did not elicit significant effects), but differed from Ponomarenko *et al*. (whose team found the CL variants to display different bindings to cyclic and linear peptide epitopes). Since our antigen is the recombinant extracellular portion of Her2, and Ponomarenko *et al*. observed differences based on the conformation of the antigen, it is possible that CL variation effects may be antibody-antigen interaction dependent^18^ and cannot be generalized to all antibodies. It is likely that the structural and rigidity of the antibody, influenced by modification in the light chain as hinted by Toughiri *et al*.^20^, may underlie the mechanism for the differences. Nonetheless, it is clear that for recombinant extracellular Her2 binding, both Trastuzumab and Pertuzumab CL variants did not show significant effects in our assays.

Currently, there remain significant hurdles for CH and CL variants of therapeutic antibodies to be adopted in actual clinical use given that pre-clinical animal experiments in this area are severely limited. Without suitable methods to assess both the potential benefits and immunopathology of antibody isotypes/subtypes as therapeutics (e.g. IgE anaphylaxis, IgA nephropathy, IgM rheumatoid factor, Hyper-IgD syndrome), it may be decades before CH isotype swapping are adopted as the next generation of antibody therapeutics.

## Conclusion

Our study has demonstrated the biophysical feasibility of full human antibody isotype swapping for therapeutic antibodies without significantly compromising antigen-binding and production (with the exception of IgD). With suitable animal or pre-human models, it is foreseeable that future antibody targeted therapies may utilize the same therapeutic antibody in varying ratios of isotype/subtype variants for improved localization and reduced systemic side effects, particularly, for the heavy chain.

## Materials and Methods

### Cloning of antibodies

VL and VH genes of Trastuzumab (PDB: 1N8Z) and Pertuzumab (PDB: 1S78) were synthesized (Blue Heron Biotech LLC). The VL were joined to Cκ (Accession no: AKL91149.1) and Cλ (Cλ1: Accession X51755; Cλ2: Accession J00253; Cλ3: Accession X06876; Cλ6: Accession J03011; Cλ7: Accession X51755) forming the light chain, while the VH were joined to CH isotype/subtypes (CHμ: Accession AAS01769.1; CHα1: Accession P01876.2; CHα2: Accession AAT74071.1; CHγ1: Accession AAA02914.1; CHγ2: Accession AAG00910.2; CHγ3: Accession P01860.2; CHγ4: Accession P01861.1; CHδ: Accession AAA52770.1; and CHε: Accession P01854.1) to form the heavy chain construct in cloning cassettes previously described^36^. The cassettes were cloned into pTT5 plasmid (YouBio) and transformed into competent DH5α bacteria^37^ as previously described.

### Production and purification of antibodies

Antibodies variants of both Trastuzumab and Pertuzumab were produced in the following combinations:

Light chain variants: CLκ/CHγ1; CLλ1/CHγ1; CLλ2/CHγ1; CLλ3/CHγ1; CLλ6/CHγ1; CLλ7/CHγ1.

Heavy chain variants: CLκ/CHμ; CLκ/CHα1; CLκ/CHα2; CLκ/CHδ; CLκ/CHγ1; CLκ/CHγ2; CLκ/CHγ3; CLκ/CHγ4; CLκ/CHε.

The antibody heavy and light chain plasmids were transfected into EXPI293F (Invitrogen, Cat no. A14527) as previously performed^38^. Briefly, the IgG variants transfected cells were cultured in DMEM with 10% low IgG FBS (Pan Biotech, Cat no. P30-2802) and purified using Protein G column while the other isotypes (IgM, IgA, IgD & IgE) were grown in DMEM with 10% FBS (Invitrogen, Cat no. 10270106) and the antibodies purified using Protein L column. Affinity chromatography purifications were carried out two weeks after transfection followed by gel filtration analysis using Superdex 200 pg 16/600 size exclusion column (GE Healthcare) to extract the suitable fractions (monomeric for all except IgM). Using the Gel filtration HMW calibration kit (GE, Cat no: 28-4038-42) and the Gel filtration LMW calibration kit (GE, Cat no: 28-4038-41), the 70 ml mark was found to correspond to ~150 kDa when using Superdex 200 pg 16/600 in our system (see supplementary date for calibration graph of protein molecular weight and elution time). Gel filtered fractions were concentrated using 100 kDa Amicon Ultra protein concentrator (Merck Millipore, Cat no. UFC910096,) and evaluated by UV analysis (Nanodrop 1000 spectrophotometer, Thermo Fisher Scientific) and with the extinction coefficient values determined from ProtParam^39^. Gel filtration figures were generated by the Unicorn 6.0 software (GE Healthcare) with lines thickened using the GIMP 2.9.4 software. Purified antibody variants were analysed on reducing 10% SDS-PAGE gels and stained using Bio-Safe Coomassie stain (Bio-Rad, Cat no. 1610786). Gel band sizes were quantified automatically using GelApp^40^ and protein lanes were crop out and attached with corresponding size exclusion chromatography graph (for full gel photos with ladder, please see Supplementary Data). All the purification steps using affinity or size exclusion chromatography were carried out automatically using the AKTA Pure system, keeping the same settings for all the antibodies used in this study.

### Binding affinity studies

Measurements of the association and dissociation rates of the antibodies were carried out using two methods. The first method was the direct binding of antibodies to either Anti-hIgG-Fc Capture (AHC) biosensors (Fortebio, Cat no. 18-5060) for light chain isotypes or Protein L (ProL) biosensors (Fortebio, Cat no. 18-5085) for heavy chain isotypes, prior to binding to Her2 at 100 nM to 6.25 nM using 1 × kinetic buffer (Fortebio, Cat no. 18-1092) on the Octet QK^e^ system (Fortebio, Cat no. 30-5046). All readings (KD, Ka and Kd) were generated using the Octet data analysis software, and graphs of represented set of 3 repeats were re-plotted using Microsoft Excel 2010. KD was calculated automatically by the software where KD = Kd/Ka. Statistical error for Ka and Kd were calculated by the software based on the replicate experiments. As the KD readings are calculated, they do not have statistical error.

The second method setup was the pre-binding of HIS-tagged Her2 (Sino Biologicals Inc, Cat no. H10004-H08H) onto the Ni-NTA (NTA) biosensors (Fortebio, Cat no. 18-5101) on the Blitz system (Fortebio, Cat no. 45-5000) as performed in Lua *et al*. using 1 × kinetic buffer^25^. Association and dissociation readings (Ka, Kd and KD) were calculated based on 3 repeats, generated using Blitz system and binding graphs were re-plotted using Microsoft Excel 2010.

### Data availability

The datasets generated during and/or analysed during the current study are available from the corresponding author on reasonable request.

## Electronic supplementary material

Supplementary Information
