# Author Correction: The effects of Antibody Engineering CH and CL in Trastuzumab and Pertuzumab recombinant models: Impact on antibody production and antigen-binding

**DOI:** 10.1038/s41598-018-29097-z

**Published:** 2018-07-18

**Authors:** Wai-Heng Lua, Wei-Li Ling, Joshua Yi Yeo, Jun-Jie Poh, David Philip Lane, Samuel Ken-En Gan

**Affiliations:** 1 Bioinformatics Institute, Agency for Science, Technology and Research (A*STAR), Singapore, Singapore; 2 p53 Laboratory, Agency for Science, Technology and Research (A*STAR), Singapore, Singapore

Correction to: *Scientific Reports* 10.1038/s41598-017-18892-9, published online 15 January 2018

This Article contains errors in the Methods section, under subsection ‘Cloning of antibodies’.

“VL and VH genes of Trastuzumab (PDB: 1N8Z) and Pertuzumab (PDB: 1S78) were synthesized (Blue Heron Biotech LLC). The VL were joined to Cκ (Accession no: AKL91149.1) and Cλ (Cλ1: Accession X51755; Cλ2: Accession J00253; Cλ3: Accession X06876; Cλ6: Accession J03011; Cλ7: Accession X51755) forming the light chain, while the VH were joined to CH isotype/subtypes (CHμ: Accession AAS01769.1; CHα1: Accession P01876.2; CHα2: Accession AAT74071.1; CHγ1: Accession AAA02914.1; CHγ2: Accession AAG00910.2; CHγ3: Accession P01860.2; CHγ4: Accession P01861.1; CHδ: Accession AAA52770.1; and CHε: Accession P01854.1) to form the heavy chain construct in cloning cassettes previously described^36^. The cassettes were cloned into pTT5 plasmid (YouBio) and transformed into competent DH5α bacteria^37^ as previously described.”

should read:

“VL and VH genes of Trastuzumab (PDB: 1N8Z) and Pertuzumab (PDB: 1S78) were synthesized (Blue Heron Biotech LLC). The VL were joined to Cκ (Accession no: AKL91149.1) and Cλ (Cλ1: Accession P0CG04.1; Cλ2: Accession P0DOY2.1; Cλ3: Accession P0DOY3.1; Cλ6: Accession P0CF74.1; Cλ7: Accession AAB59435.1) forming the light chain, while the VH were joined to CH isotype/subtypes (CHμ: Accession AAS01769.1; CHα1: Accession P01876.2; CHα2: Accession AAT74071.1; CHγ1: Accession AAA02914.1; CHγ2: Accession AAG00910.2; CHγ3: Accession P01860.2; CHγ4: Accession P01861.1; CHδ: Accession AAA52770.1; and CHε: Accession P01854.1) to form the heavy chain construct in cloning cassettes previously described ^36^. The cassettes were cloned into pTT5 plasmid (YouBio) and transformed into competent DH5α bacteria^37^ as previously described.”

