# Supplementary material for: The effects of Antibody Engineering CH and CL in Trastuzumab and Pertuzumab recombinant models: Impact on antibody production and antigen-binding
Source: Sci Rep. 2018 Jan 15;8:718. doi: 10.1038/s41598-017-18892-9 (PMC5768722; doi:10.1038/s41598-017-18892-9)
Supplement: Supplementary file 1 — Supplementary Information [file 41598_2017_18892_MOESM1_ESM.pdf]

The effects of Antibody Engineering CH and CL in Trastuzumab and Pertuzumab recombinant models: Impact on antibody production and antigen-binding

Wai-Heng Lua[1], Wei-Li Ling[1], Joshua Yi Yeo[1], Jun-Jie Poh[1], David Philip Lane[2], Samuel Ken-En Gan[1,2,*]

Affiliation:

[1]Bioinformatics Institute, Agency for Science, Technology and Research (A*STAR), Singapore

[2]p53 Laboratory, Agency for Science, Technology and Research (A*STAR), Singapore

*Corresponding author: samuelg@bii.a-star.edu.sg

Bioinformatics Institute, A*STAR

30 Biopolis Street, #07-01 Matrix

Singapore 138671

Tel: +65 6407 0584

Fax : +65 6478 9047

Email addresses of authors (from left to right): luawh@bii.a-star.edu.sg; lingwl@bii.a-star.edu.sg; bii-apdlab@bii.a-star.edu.sg; pohjj@bii.a-star.edu.sg; dplane@p53lab.a-star.edu.sg; samuelg@bii.a-star.edu.sg

Keywords: Antibody class-switching, Isotype, Pertuzumab, Trastuzumab, Therapeutic antibodies.

Size Exclusion Chromatography fractionations

The calibration of the size exclusion chromatography experiments were performed using the Superdex 200pg 16/600 (GE Healthcare) of the AKTA PURE system. The calibrations were carried out using the Gel filtration Low Molecular Weight (LWM) and High Molecular Weight (HMW) calibration kits (Catalogue: 28-4038-41 & 28-4038-42). The graph, generated using Unicorn v6.3 (GE Healthcare) was plotted as per manufacturer's recommendations.

Certain protein standards in the LMW and HMW overlapped, and were used to generate the full spectrum of the elution times of the various molecular weights (Figure S1a).

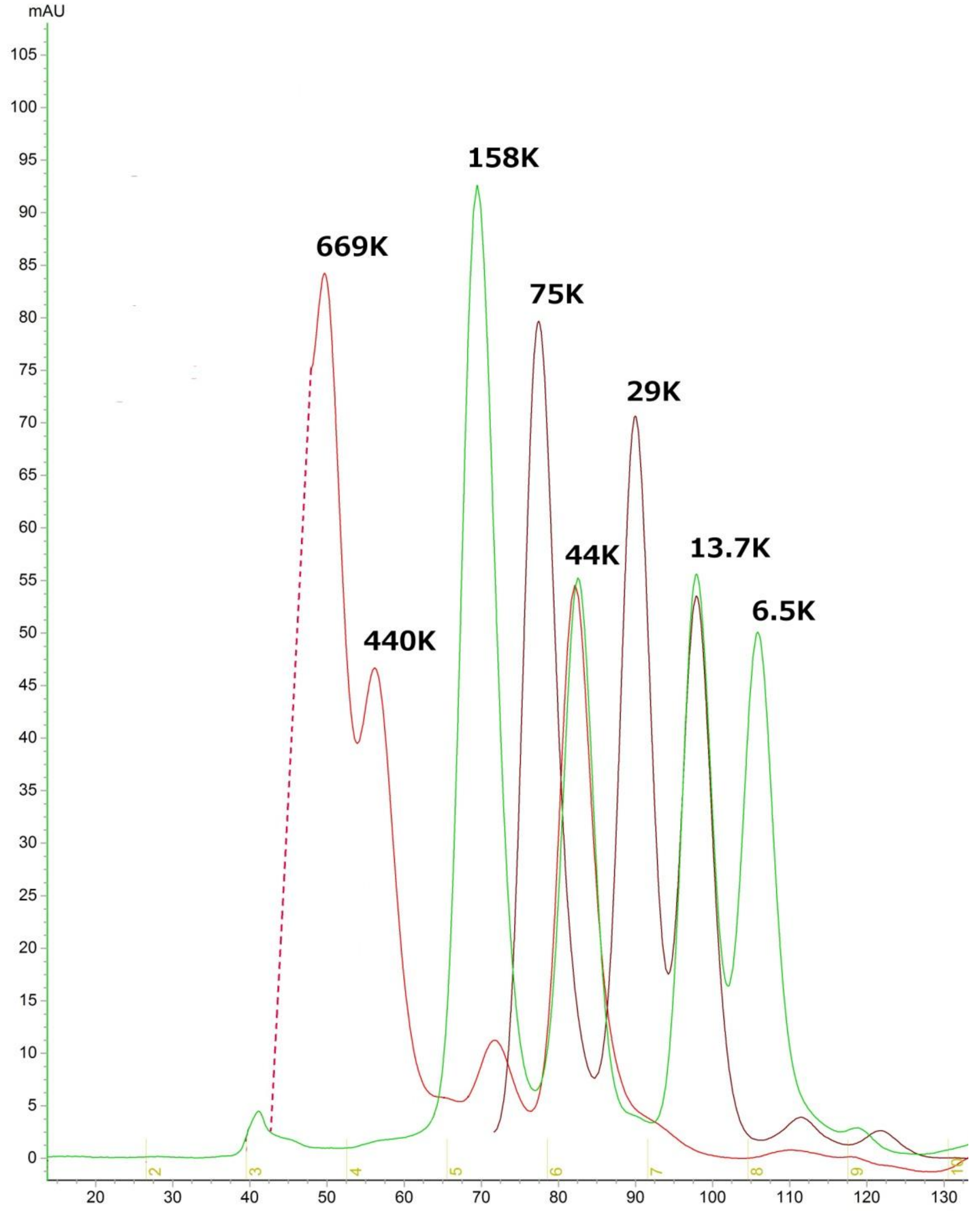

Figure S1a: Calibration curve of the various molecular weight standards in kDa.

Calibration of size exclusion chromatography on Superdex 200pg 16/600 (GE Healthcare) was performed using the AKTA PURE system with the Gel filtration Low Molecular Weight (LWM) and High Molecular Weight (HMW) calibration kits (Catalogue: 28-4038-41 & 28-4038-42). The calibration graph, generated using Unicorn v6.3 (GE Healthcare) was plotted as per manufacturer's recommendations. Both the spectrums from the protein standards in the LMW and HMW were pieced together to generate the full spectrum of the elution times of the various molecular weights.

X-Axis: 40-100 ml time scale. Y-axis: mAU absorption as determined by UV detection.

1) Trastuzumab variants

For IgM, fraction 2 was collected while for IgA, IgE, and IgD, fractions 3 and 4 were collected. For other IgGs, the various fractions are shown below. Figure S1 and S2 are used for figure 1 while figure S3 and S4 are used for figure 4.

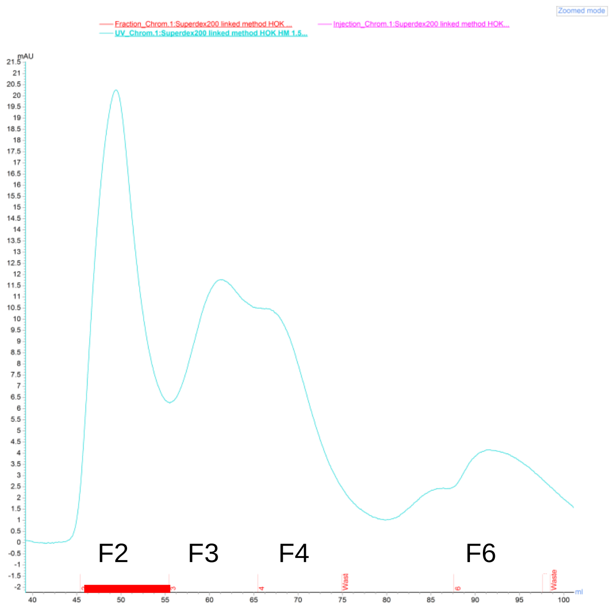

Figure S1b: Trastuzumab IgM

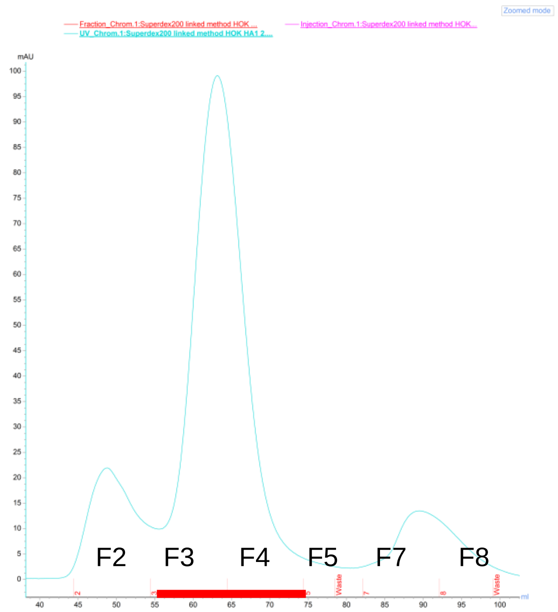

Figures S1c: Trastuzumab IgA1

Figure S1b and S1c shows the size exclusion chromatogram profiles of Trastuzumab IgM and IgA1. The collected fractions were indicated by the bold red line. Fractions corresponding to the molecular weight of pentameric/hexameric forms of IgM were collected, whereas the fractions pertaining to the monomeric fractions of IgA were collected.

X-Axis: 40-100 ml time scale. Y-axis: mAU absorption as determined by UV detection.

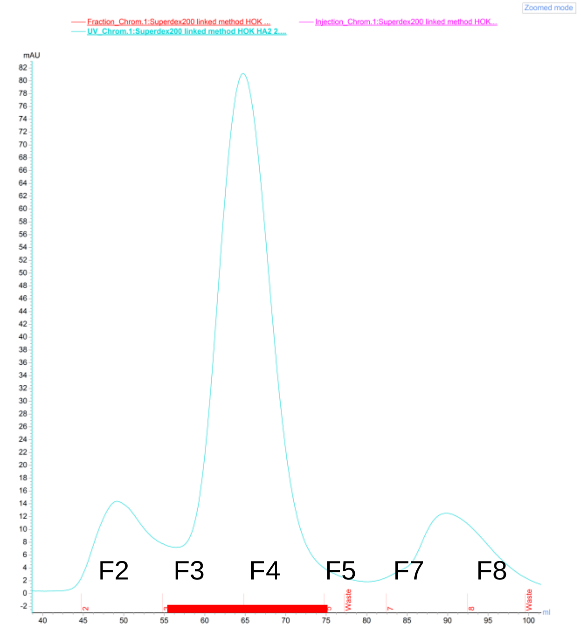

Figure S1d: Trastuzumab IgA2

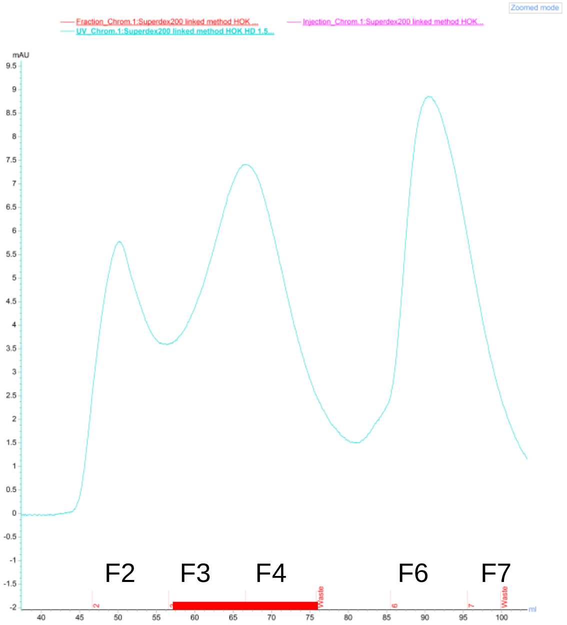

Figure S1e: Trastuzumab IgD

Figure S1d and S1e shows the size exclusion chromatogram profiles of IgA2 and IgD. Only the fractions corresponding to the ~150 kDa monomeric fractions were collected as indicated by the bold red line on the X-axis.

X-Axis: 40-100 ml time scale. Y-axis: mAU absorption as determined by UV detection.

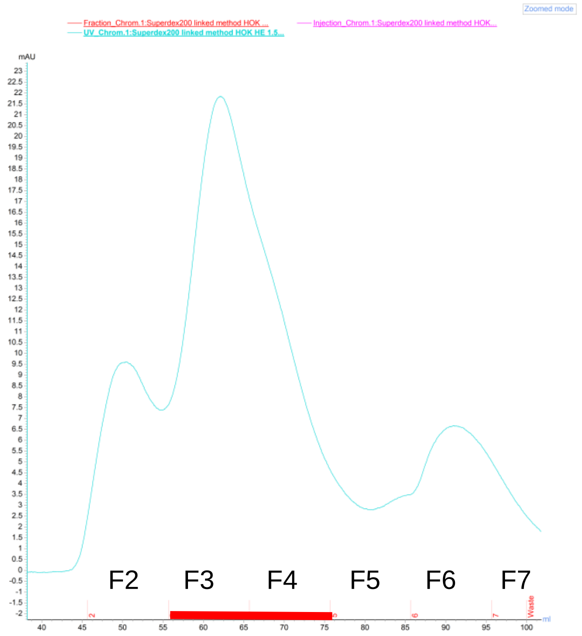

Figure S1f: Trastuzumab IgE

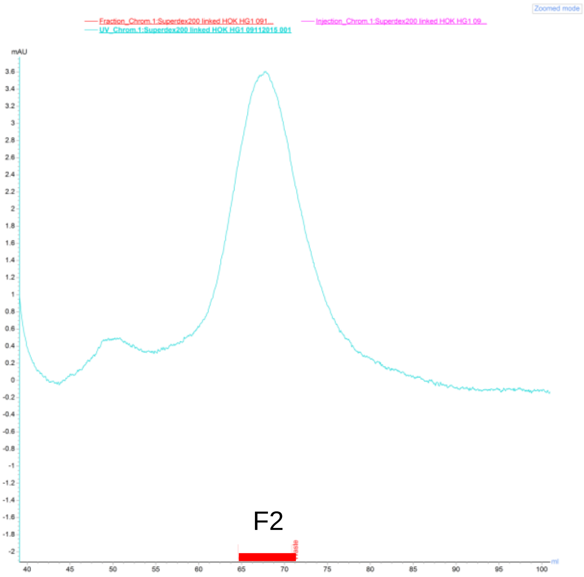

Figure S1g: Trastuzumab IgG1

Figure S1f and S1g shows the size exclusion chromatogram profiles of Trastuzumab IgE and IgG1. Only the fractions corresponding to the ~150 kDa monomeric fractions were collected as indicated by the bold red line on the X-axis.

X-Axis: 40-100 ml time scale. Y-axis: mAU absorption as determined by UV detection.

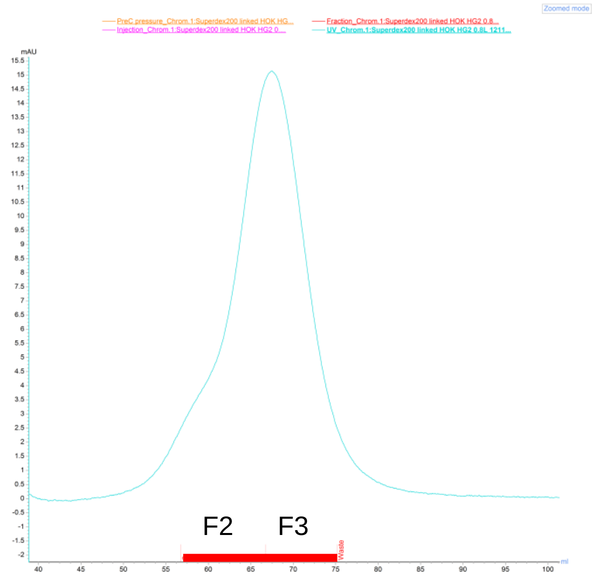

Figure S1h: Trastuzumab IgG2

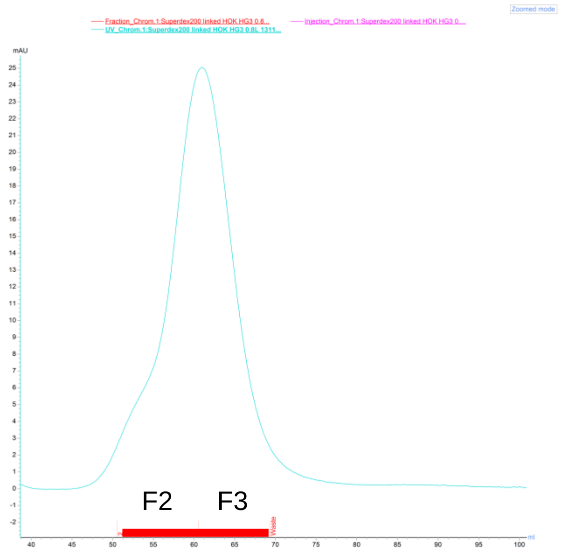

Figure S1i: Trastuzumab IgG3

Figure S1h and S1i shows the size exclusion chromatogram profiles of Trastuzumab IgG2 and IgG3. Only the fractions corresponding to the ~150 kDa monomeric fractions were collected as indicated by the bold red line on the X-axis.

X-Axis: 40-100 ml time scale. Y-axis: mAU absorption as determined by UV detection.

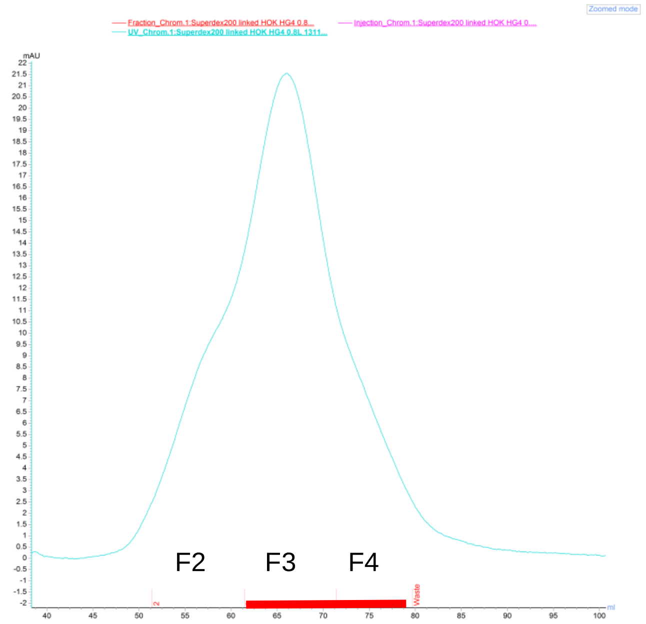

Figure S1j: Trastuzumab IgG4

Figure S1j shows the size exclusion chromatogram profile of Trastuzumab IgG4. Only the fractions corresponding to the ~150 kDa monomeric fractions were collected as indicated by the bold red line on the X-axis.

X-Axis: 40-100 ml time scale. Y-axis: mAU absorption as determined by UV detection.

2) Pertuzumab variants

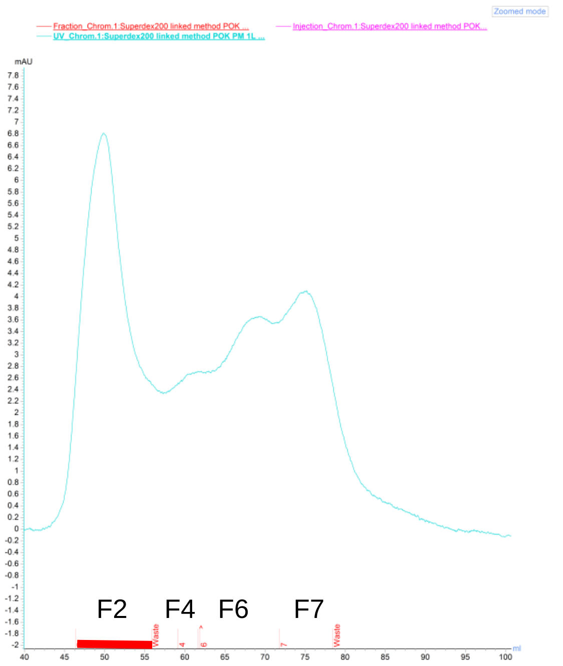

Figure S2a: Pertuzumab IgM

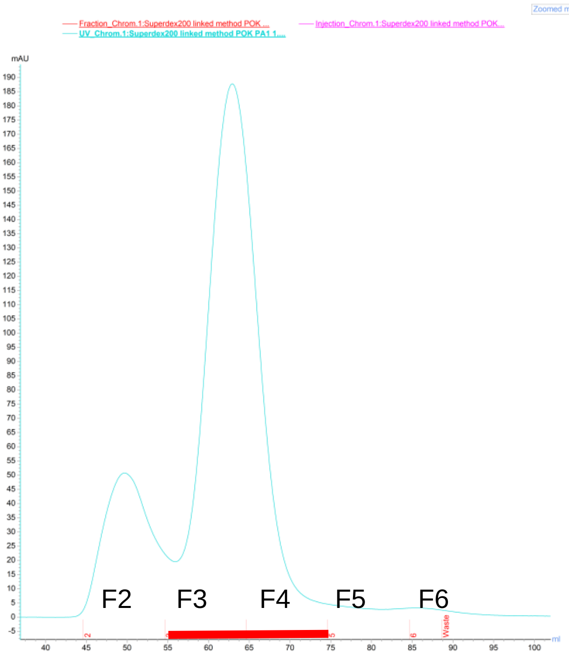

Figure S2b: Pertuzumab IgA1

Figure S2a and S2b shows the size exclusion chromatogram profiles of Pertuzumab IgM and IgA1. The collected fractions were indicated by the bold red line. Fractions corresponding to the molecular weight of pentameric/hexameric forms of IgM were collected, whereas the fractions pertaining to the monomeric fractions of IgA were collected.

X-Axis: 40-100 ml time scale. Y-axis: mAU absorption as determined by UV detection.

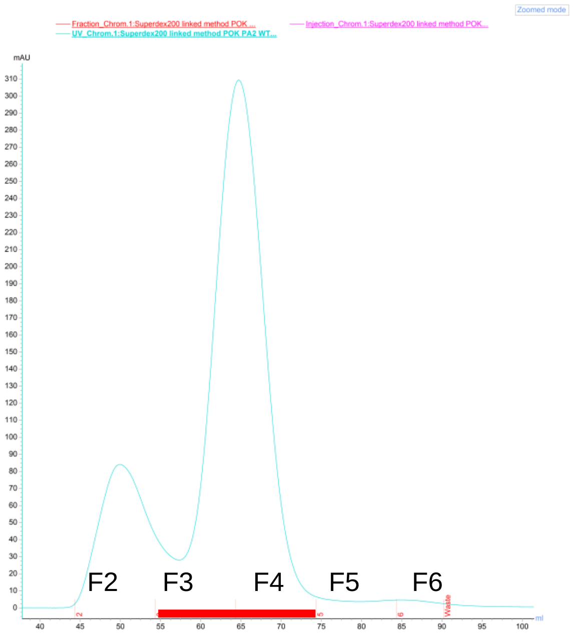

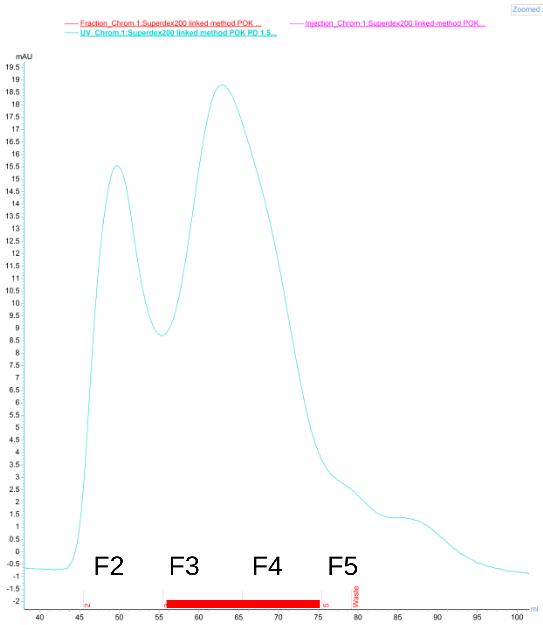

Figure S2c: Pertuzumab IgA2 Figures S2d: Pertuzumab IgD

Figure S2c and S2d shows the size exclusion chromatogram profiles of Pertuzumab IgA2 and IgD. Only the fractions corresponding to the ~150 kDa monomeric fractions were collected as indicated by the bold red line on the X-axis.

X-Axis: 40-100 ml time scale. Y-axis: mAU absorption as determined by UV detection.

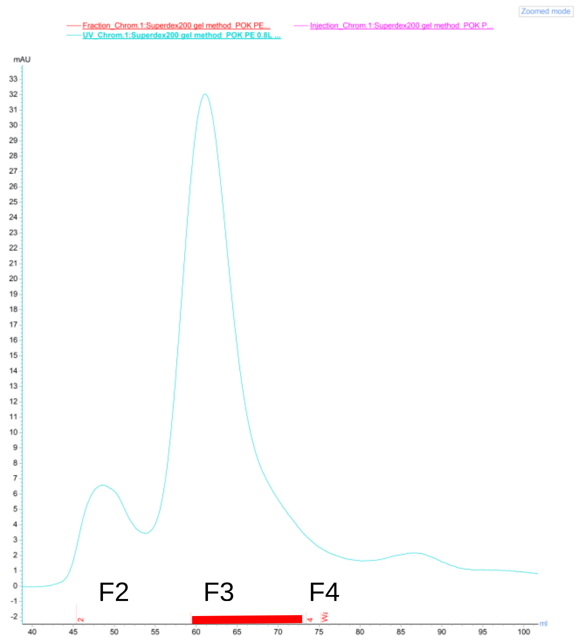

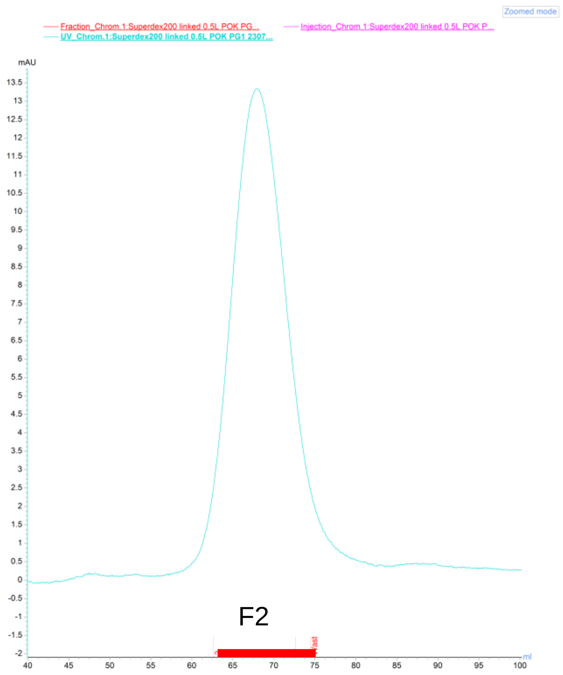

Figure S2e: Pertuzumab IgE Figure S2f: Pertuzumab IgG1

Figure S2e and S2f shows the size exclusion chromatogram profiles of Pertuzumab IgE and IgG1. Only the fractions corresponding to the ~150 kDa monomeric fractions were collected as indicated by the bold red line on the X-axis.

X-Axis: 40-100 ml time scale. Y-axis: mAU absorption as determined by UV detection.

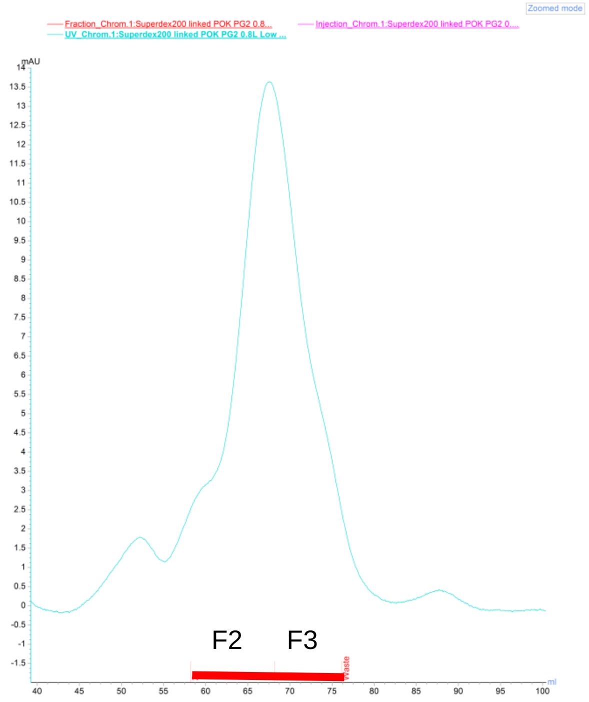

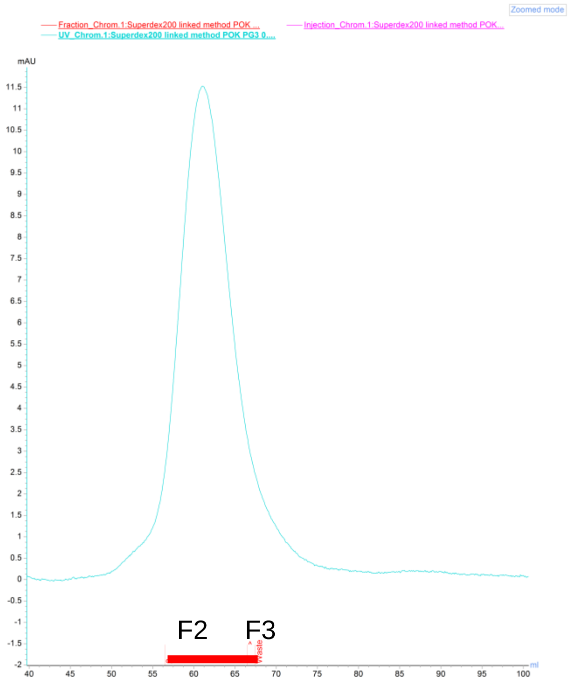

Figure S2g: Pertuzumab IgG2

Figure S2h: Pertuzumab IgG3

Figure S2g and S2h shows the size exclusion chromatogram profiles of Pertuzumab IgG2 and IgG3. Only the fractions corresponding to the ~150 kDa monomeric fractions were collected as indicated by the bold red line on the X-axis.

X-Axis: 40-100 ml time scale. Y-axis: mAU absorption as determined by UV detection.

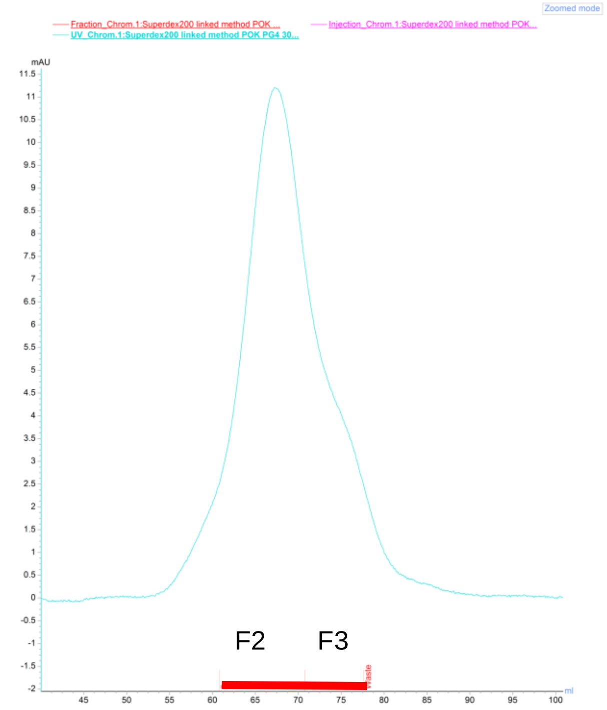

Figure S2i: Pertuzumab IgG4

Figure S2i shows the size exclusion chromatogram profiles of Pertuzumab IgG4. Only the fractions corresponding to the ~150 kDa monomeric fractions were collected as indicated by the bold red line on the X-axis.

X-Axis: 40-100 ml time scale. Y-axis: mAU absorption as determined by UV detection.

3) λ Trastuzumab IgG1 variants

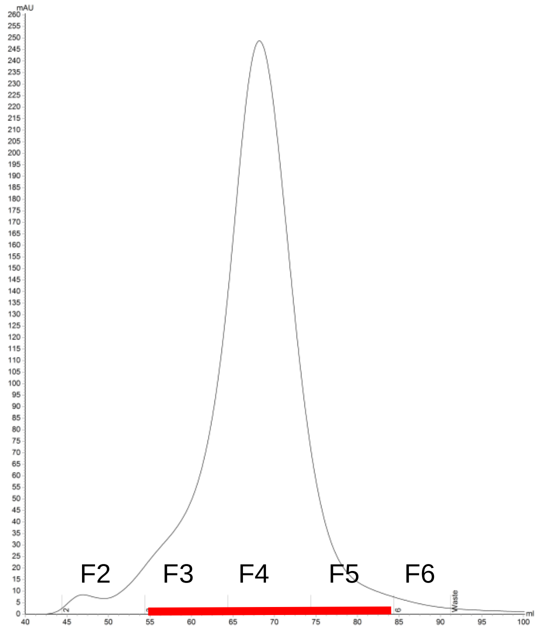

Figure S3a: Trastuzumab Cλ1 IgG1

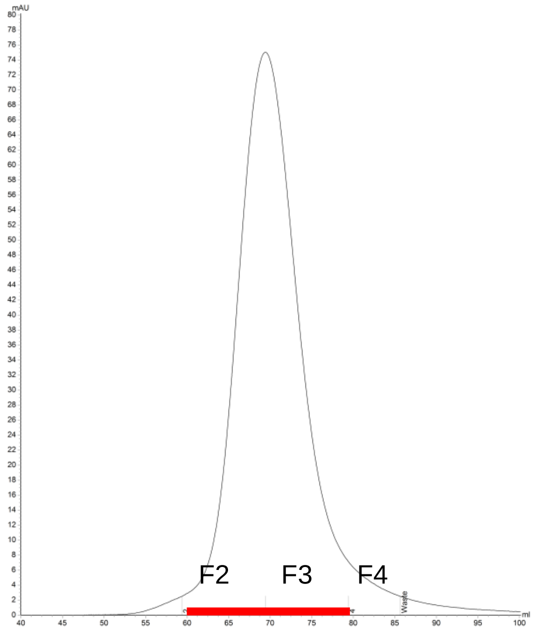

Figure S3b: Trastuzumab Cλ2 IgG1

Figure S3a and S3b shows the size exclusion chromatogram profiles of λ Trastuzumab IgG1 variants, Cλ1 and Cλ2 monomeric fraction. Only the fractions corresponding to the ~150 kDa monomeric fractions were collected as indicated by the bold red line on the X-axis.

X-Axis: 40-100 ml time scale. Y-axis: mAU absorption as determined by UV detection.

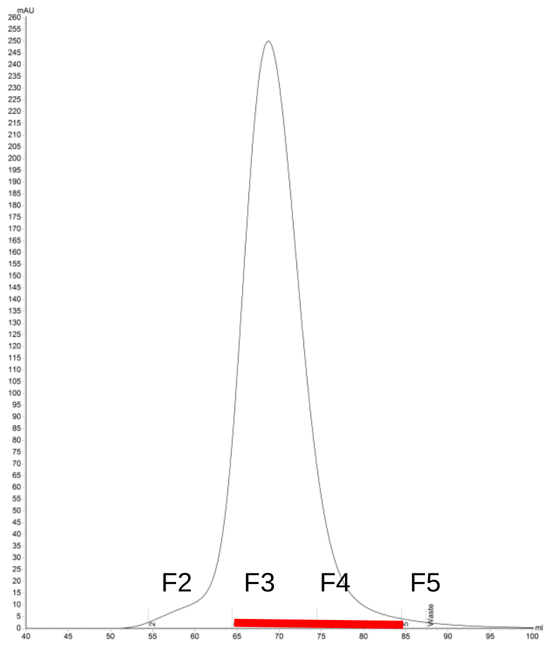

Figure S3c: Trastuzumab Cλ3 IgG1

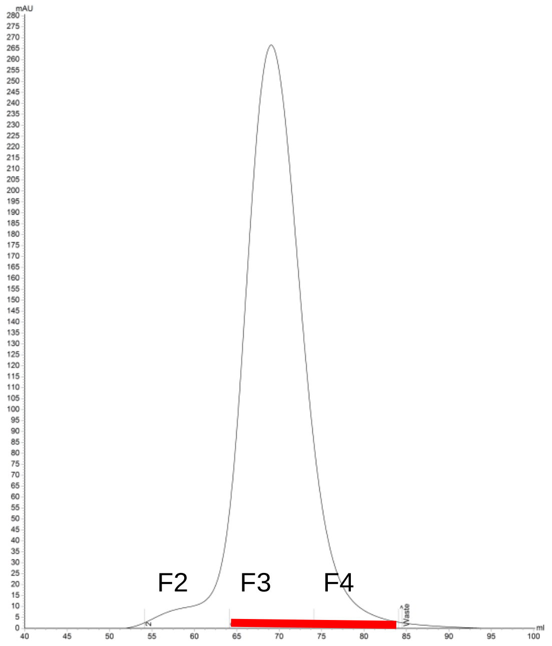

Figure S3d: Trastuzumab Cλ6 IgG1

Figure S3c and S3d shows the size exclusion chromatogram profiles of λ Trastuzumab IgG1 variants, Cλ3 and Cλ6 monomeric fraction. Only the fractions corresponding to the ~150 kDa monomeric fractions were collected as indicated by the bold red line on the X-axis.

X-Axis: 40-100 ml time scale. Y-axis: mAU absorption as determined by UV detection.

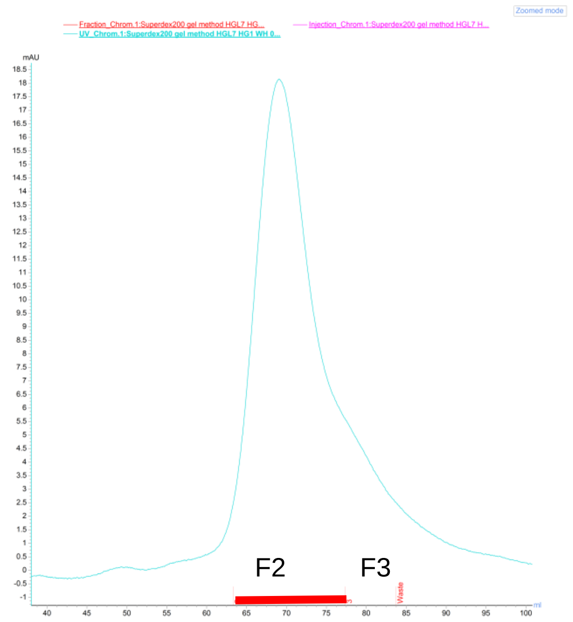

Figure S3e: Trastuzumab Cλ7 IgG1

Figure S3e shows the size exclusion chromatogram profiles of λ Trastuzumab IgG1 variants, Cλ7 monomeric fraction. Only the fractions corresponding to the ~150 kDa monomeric fractions were collected as indicated by the bold red line on the X-axis.

X-Axis: 40-100 ml time scale. Y-axis: mAU absorption as determined by UV detection.

4) λ Pertuzumab IgG1 variants

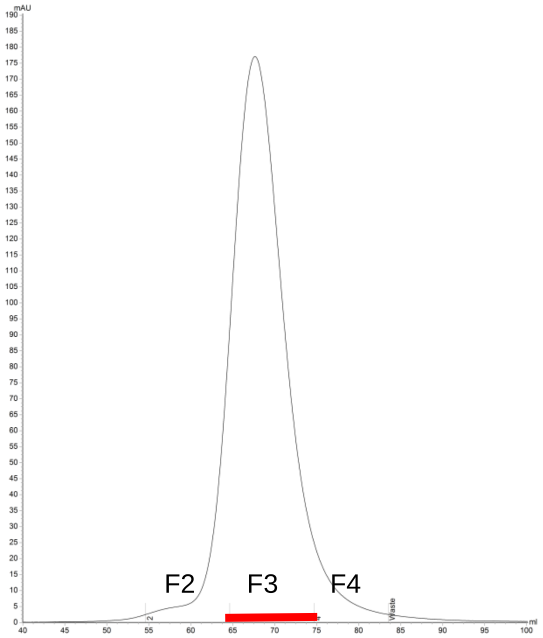

Figure S4a: Pertuzumab Cλ1 IgG1

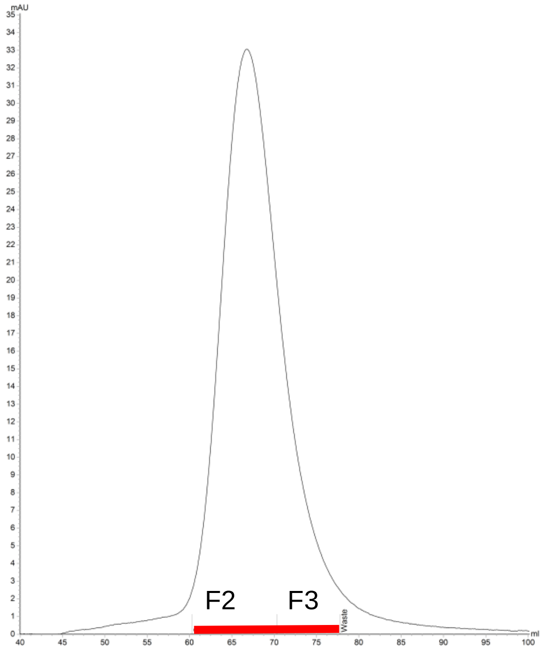

Figure S4b: Pertuzumab Cλ2 IgG1

Figure S4a and S4b shows the size exclusion chromatogram profiles of λ Pertuzumab IgG1 variants, Cλ1 and Cλ2 monomeric fraction. Only the fractions corresponding to the ~150 kDa monomeric fractions were collected as indicated by the bold red line on the X-axis.

X-Axis: 40-100 ml time scale. Y-axis: mAU absorption as determined by UV detection.

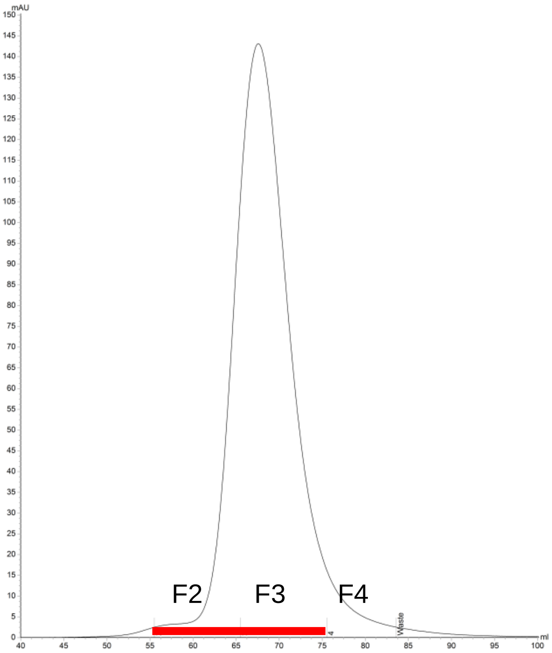

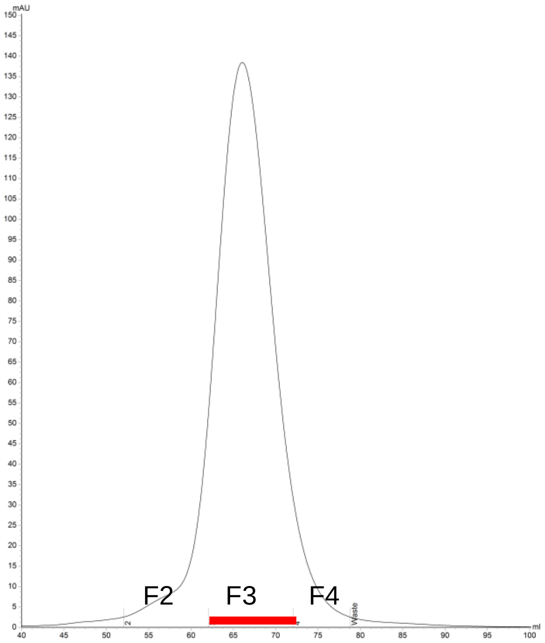

Figure S4c: Pertuzumab Cλ3 IgG1 Figure S4d: Pertuzumab Cλ6 IgG1

Figure S4c and S4d shows the size exclusion chromatogram profiles of λ Pertuzumab IgG1 variants, Cλ3 and Cλ6 monomeric fraction. Only the fractions corresponding to the ~150 kDa monomeric fractions were collected as indicated by the bold red line on the X-axis.

X-Axis: 40-100 ml time scale. Y-axis: mAU absorption as determined by UV detection.

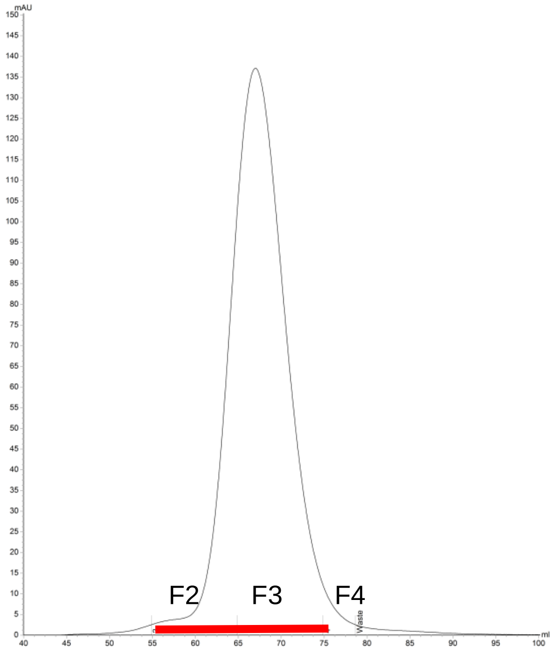

Figure S4e: Pertuzumab Cλ7 IgG1

Figure S4e shows the size exclusion chromatogram profiles of λ Pertuzumab IgG1 variants, Cλ7. Only the fractions corresponding to the ~150 kDa monomeric fractions were collected as indicated by the bold red line on the X-axis.

X-Axis: 40-100 ml time scale. Y-axis: mAU absorption as determined by UV detection.

Fraction numbers are indicated the X-axis with the bold red line and numbers. All samples were analysed using the same settings in AKTA Pure.

The x-axis is the ml (volume) of the elution segment of the SEC, based on 1ml/min flowrate on the column. Y-axis shows the mAU of the protein absorbance of UV detection.

All figures were exported and generated from the Unicorn v6 software.

SDS-PAGE analysis of the monomeric fractions of the IgA, D, G, E and oligomeric fraction of the IgM variants of Trastuzumab and Pertuzumab

SDS-PAGE gel photos of the concentrated isolated desired fractions of the antibodies.

The proteins were reduced and boiled before loading on 10%SDS-PAGE. The band sizes were determined using GelApp (Nature Methods Application Notes. doi:10.1038/an9643) with the calculated size (KDa) shown. Sections 1 and 2 show Gelapp screenshots for heavy chain variants of Trastuzumab and Pertuzumab (corresponding to figure 1 of the manuscript) while sections 3 and 4 are the λ light chain variants of Trastuzumab and Pertuzumab (corresponding to Figure 4 of the manuscript).

5) Trastuzumab variants

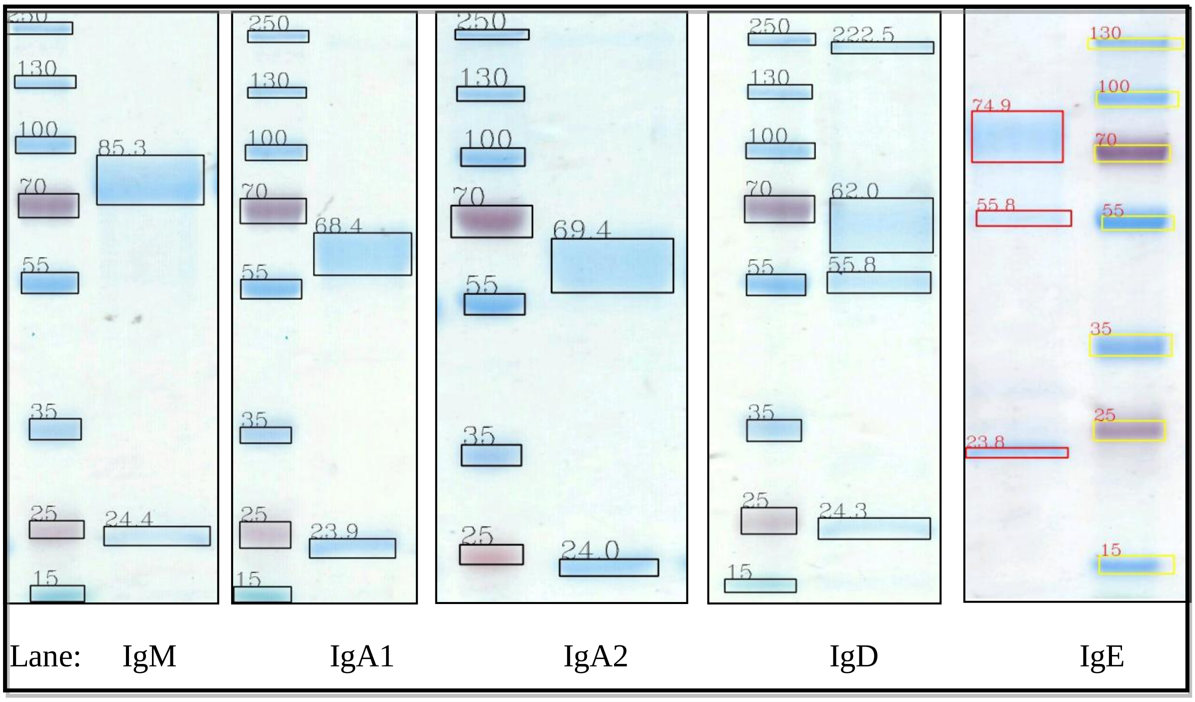

Figure S5a: SDS-PAGE of Trastuzumab isotype variants

Figure S5a shows the reducing SDS gel with the heavy and light chain band sizes of Trastuzumab IgM, IgA1, IgA2, IgD and IgE from left to right.

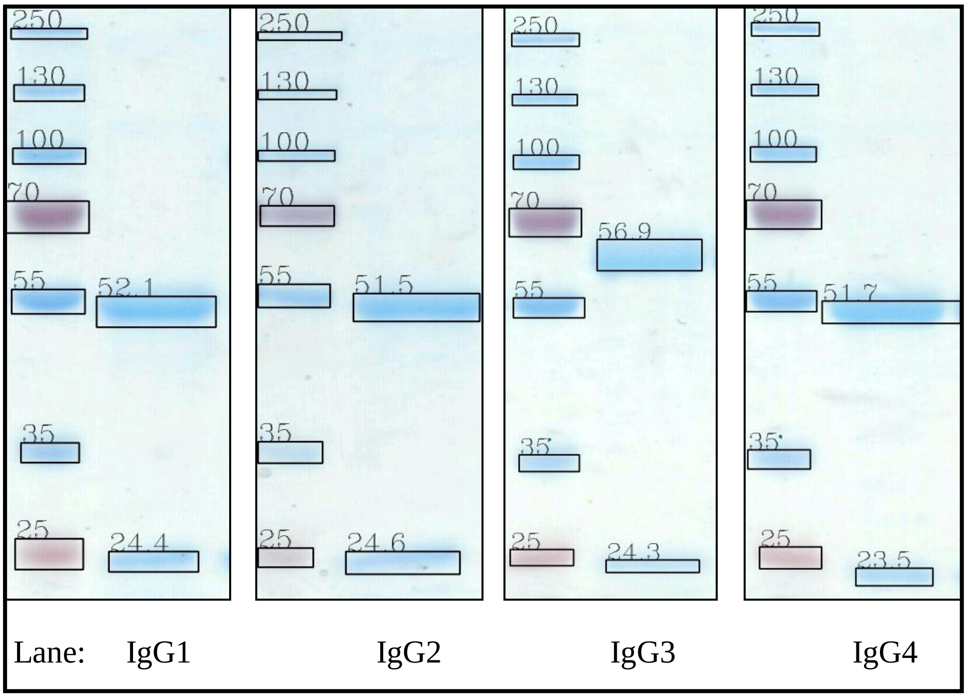

Figure S5b: SDS-PAGE of Trastuzumab isotype variants

Figure S5b shows the reducing SDS gel with the heavy and light chain band sizes of Trastuzumab IgG1, IgG2, IgG3, and IgG4 left to right.

6) Pertuzumab variants

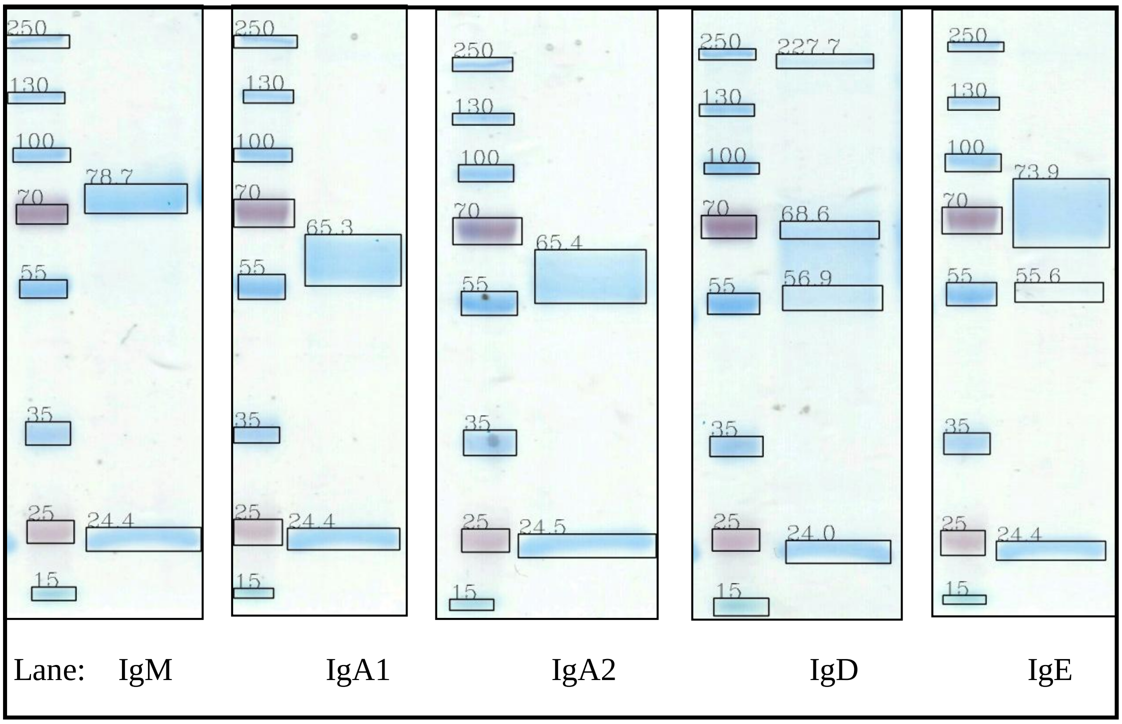

Figure S6a: SDS-PAGE of Pertuzumab isotype variants

Figure S6a shows the reducing SDS gel with the heavy and light chain band sizes of Pertuzumab IgM, IgA1, IgA2, IgD and IgE left to right.

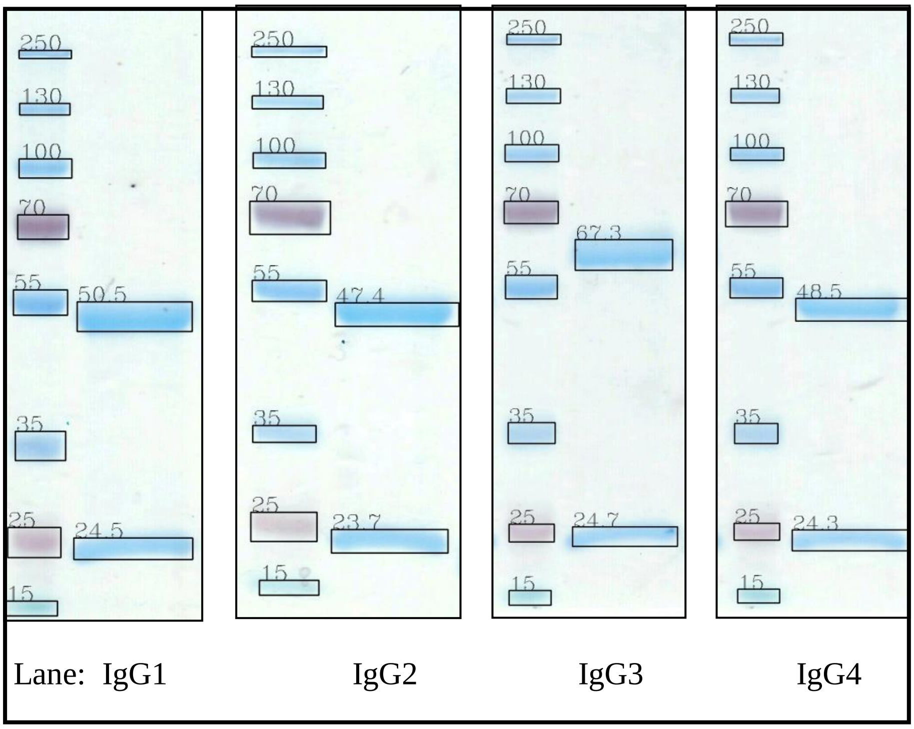

Figure S6b: SDS-PAGE of Pertuzumab isotype variants

Figure S6b shows the reducing SDS gel with the heavy and light chain band sizes of Pertuzumab IgG1, IgG2, IgG3, and IgG4 from left to right.

7) λ Trastuzumab IgG1 variants

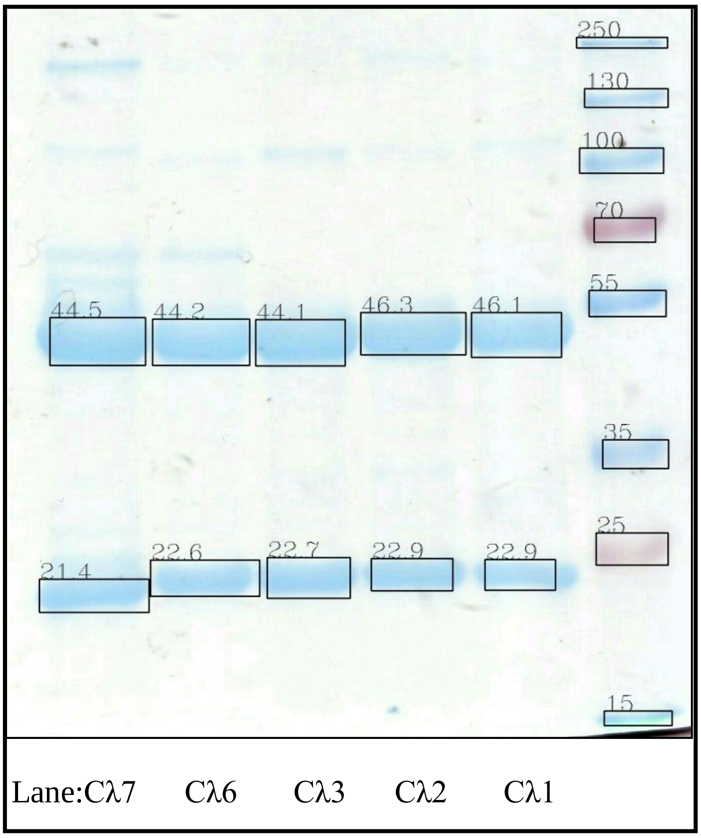

Figure S7: SDS-PAGE of λ Trastuzumab isotype variants

Figure S7 shows the reducing SDS gel with the heavy and light chain band sizes of λ Trastuzumab IgG1 variants Cλ7, Cλ6, Cλ3, Cλ2, and Cλ1 from left to right.

8) λ Pertuzumab IgG1 variants

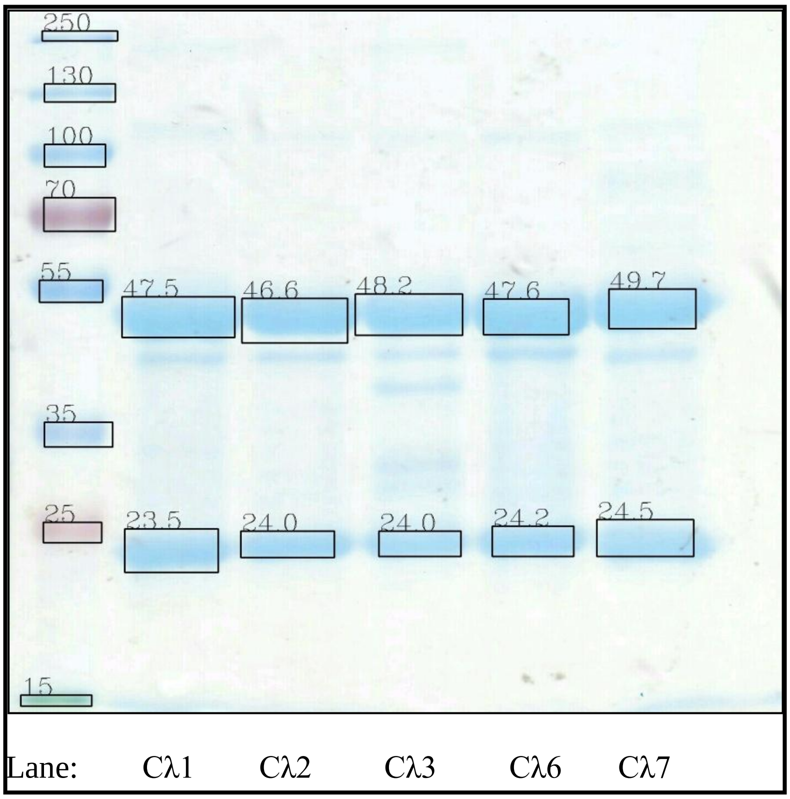

Figure S8: SDS-PAGE of λ Pertuzumab isotype variants

Figure S8 shows the reducing SDS gel with the heavy and light chain band sizes of λ Pertuzumab IgG1 variants Cλ1, Cλ2, Cλ3, Cλ6, and Cλ7 from left to right.

Affinity Purification Chromatography fractionation

The affinity purification chromatography experiments were performed using the Protein G 5ml column (GE Healthcare) or Protein L 5ml column (GE Healthcare) in the AKTA PURE system. All IgGs were pull down using Protein G column while all other isotypes were pull down using Protein L column. The elutions (as shown in red bar) were directly captured to a superloop prior to size exclusion chromatogram. The graphs were generated using Unicorn v6.3 (GE Healthcare) and plotted as per manufacturer's recommendations.

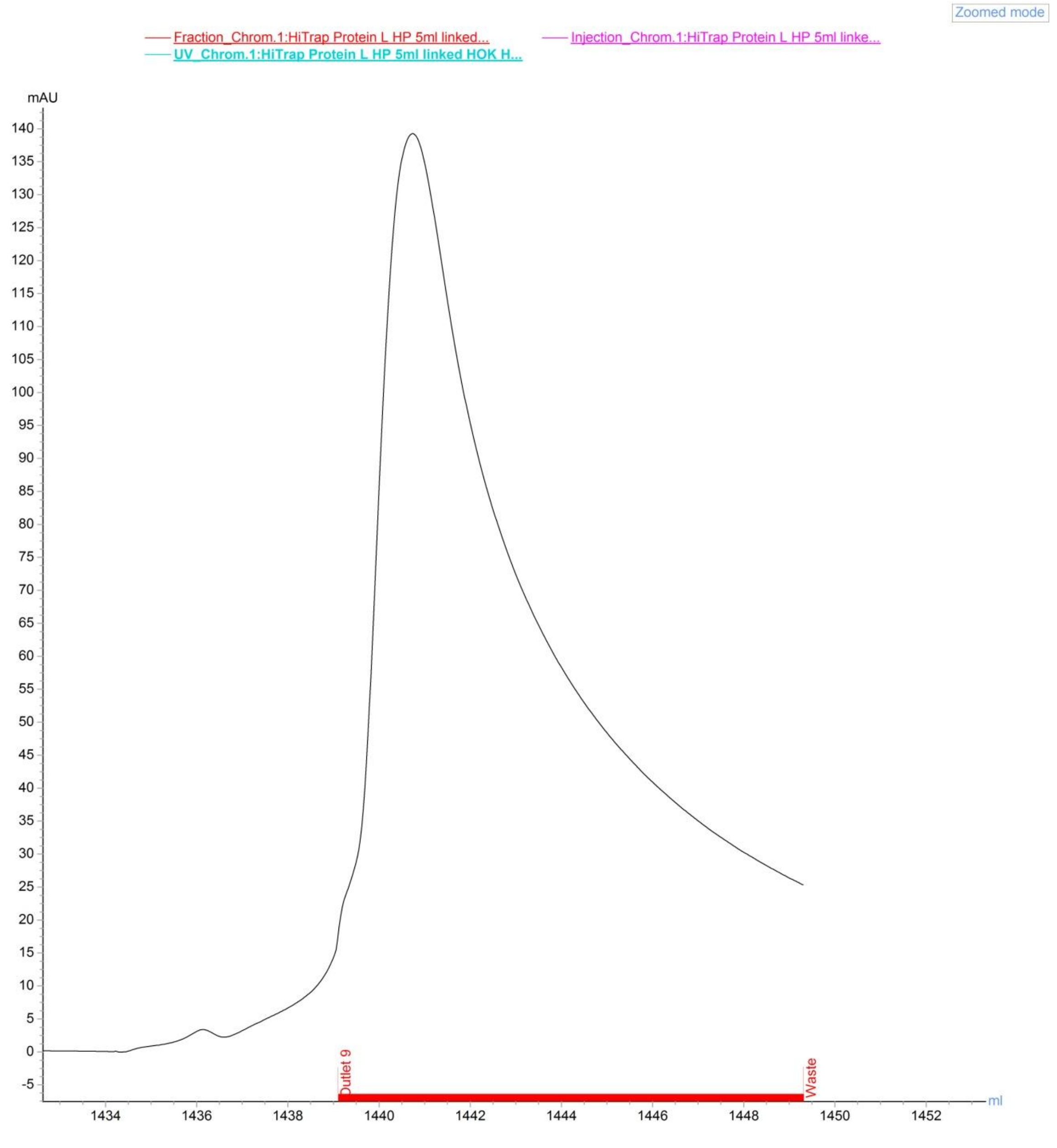

Figure S9a: Trastuzumab IgM

Figure S9a shows the affinity purification chromatogram profiles of IgM using Protein L column. Only the fractions corresponding to mAU >20 were collected as indicated by the bold red line on the X-axis.

X-Axis: ml time scale. Y-axis: mAU absorption as determined by UV detection.

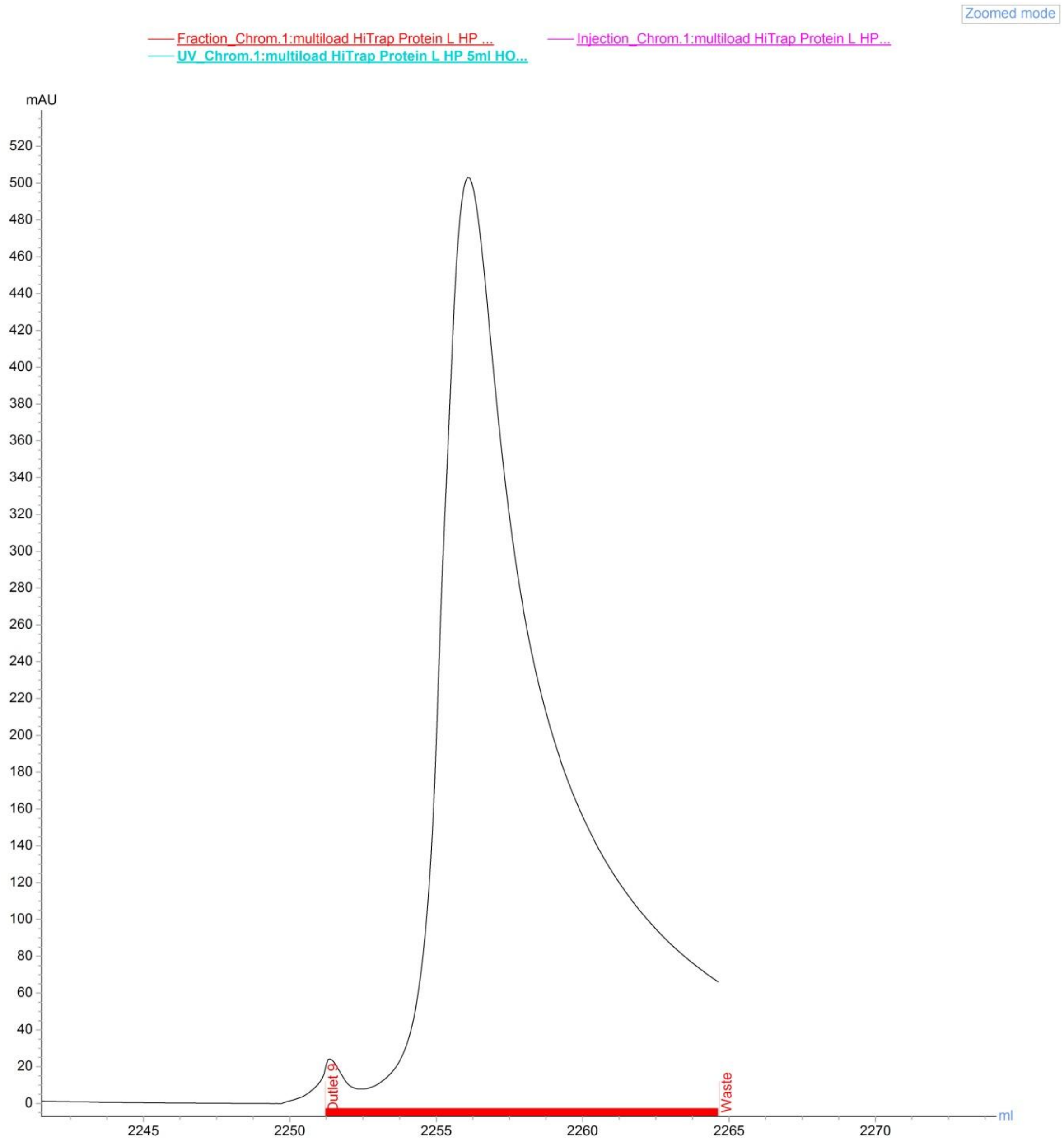

Figure S9b: Trastuzumab IgA1

Figure S9b shows the affinity purification chromatogram profiles of IgA1 using Protein L column. Only the fractions corresponding to mAU >20 were collected as indicated by the bold red line on the X-axis.

X-Axis: ml time scale. Y-axis: mAU absorption as determined by UV detection.

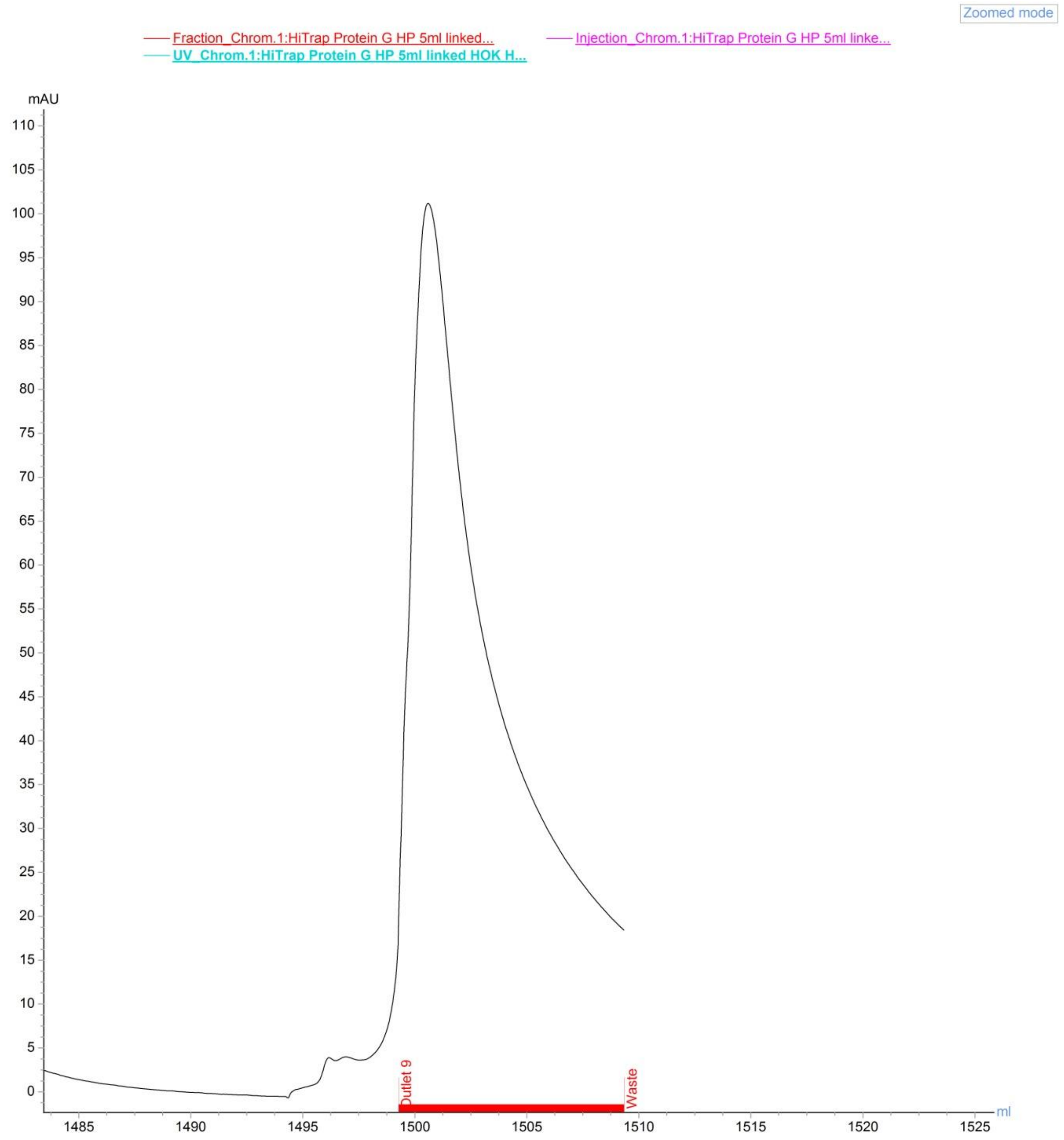

Figure S9c: Trastuzumab IgA2

Figure S9c shows the affinity purification chromatogram profiles of IgA2 using Protein L column. Only the fractions corresponding to mAU >20 were collected as indicated by the bold red line on the X-axis.

X-Axis: ml time scale. Y-axis: mAU absorption as determined by UV detection.

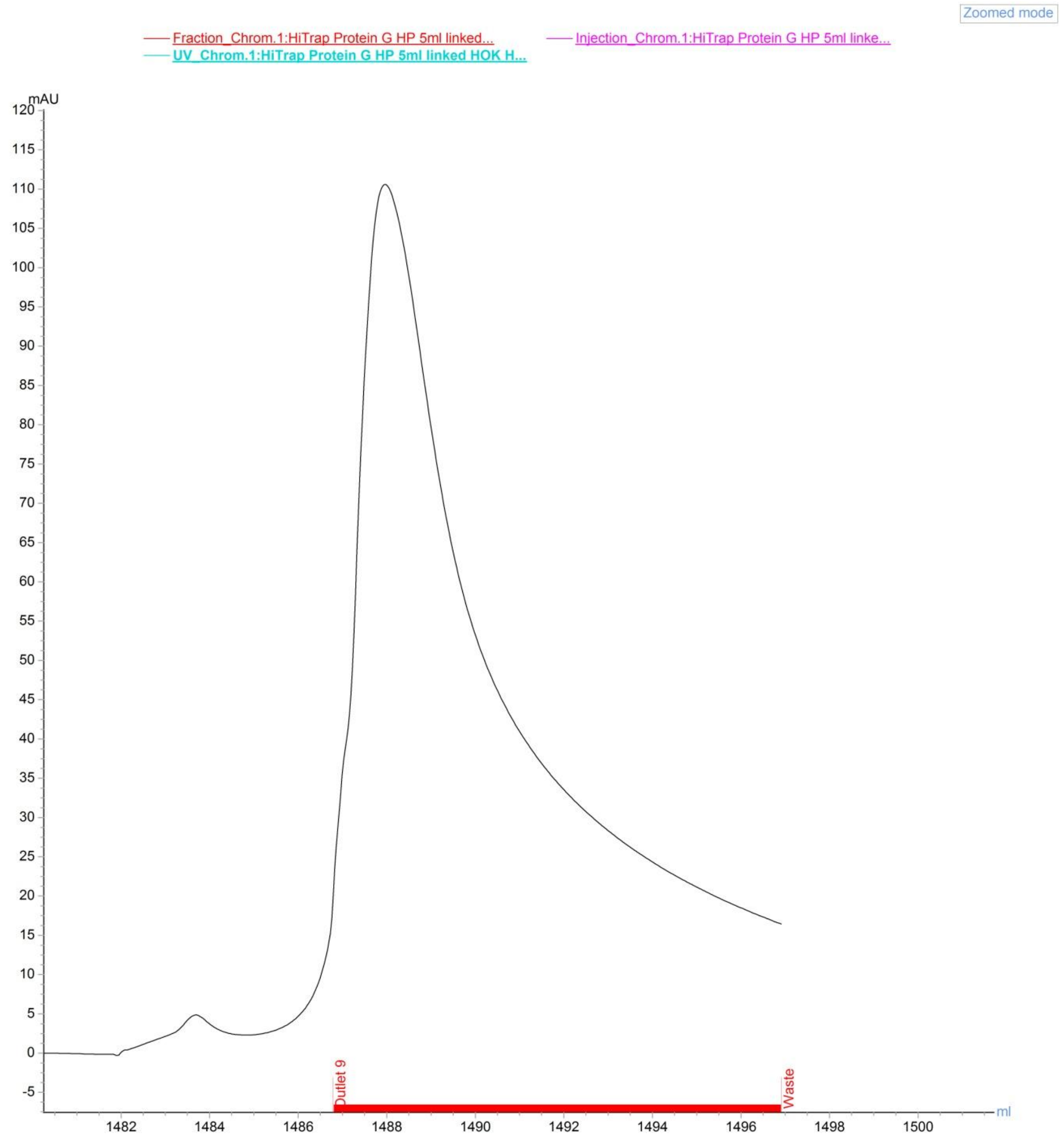

Figure S9d: Trastuzumab IgD

Figure S9d shows the affinity purification chromatogram profiles of IgD using Protein L column. Only the fractions corresponding to mAU >20 were collected as indicated by the bold red line on the X-axis.

X-Axis: ml time scale. Y-axis: mAU absorption as determined by UV detection.

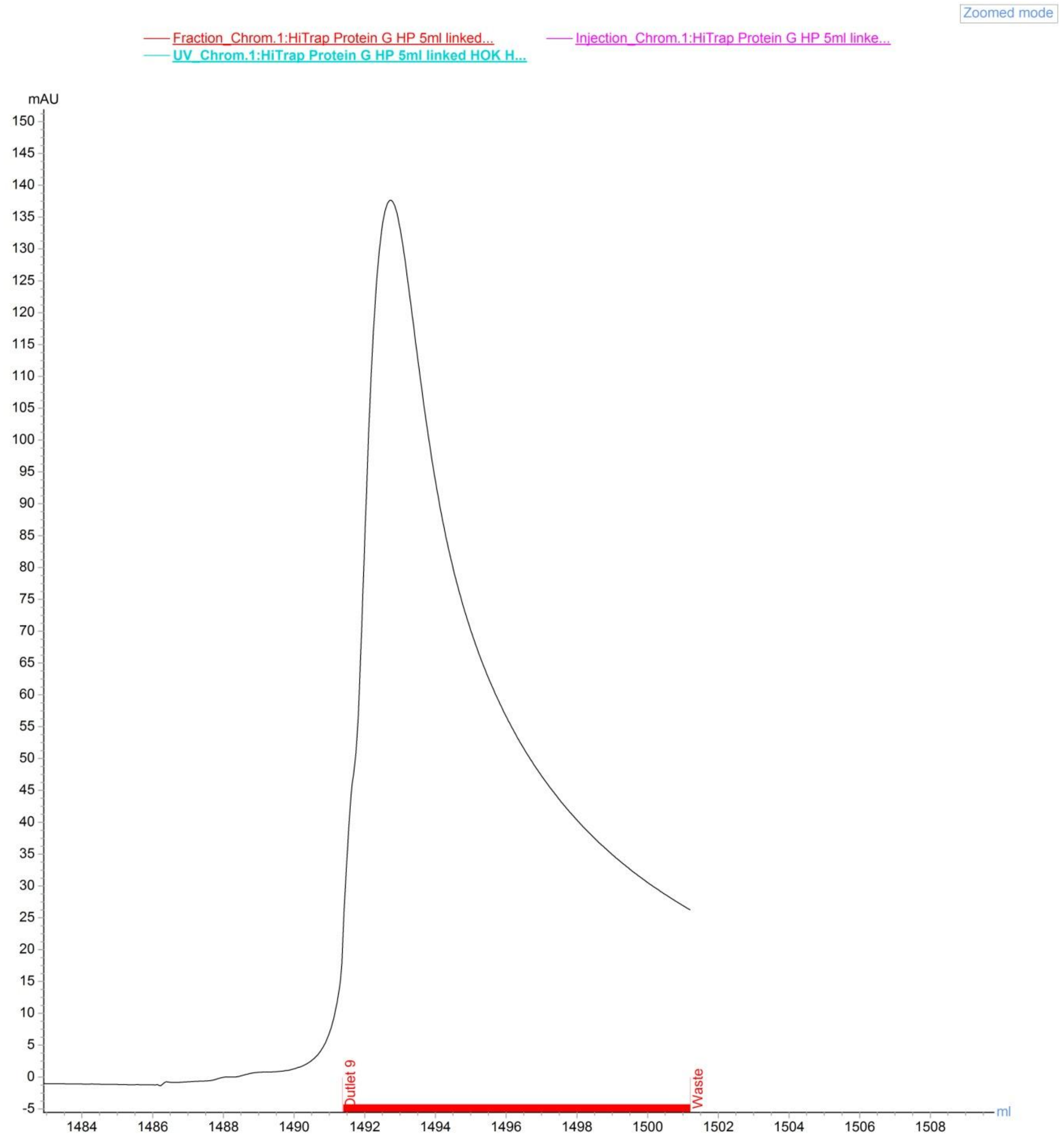

Figure S9e: Trastuzumab IgE

Figure S9e shows the affinity purification chromatogram profiles of IgE using Protein L column. Only the fractions corresponding to mAU >20 were collected as indicated by the bold red line on the X-axis.

X-Axis: ml time scale. Y-axis: mAU absorption as determined by UV detection.

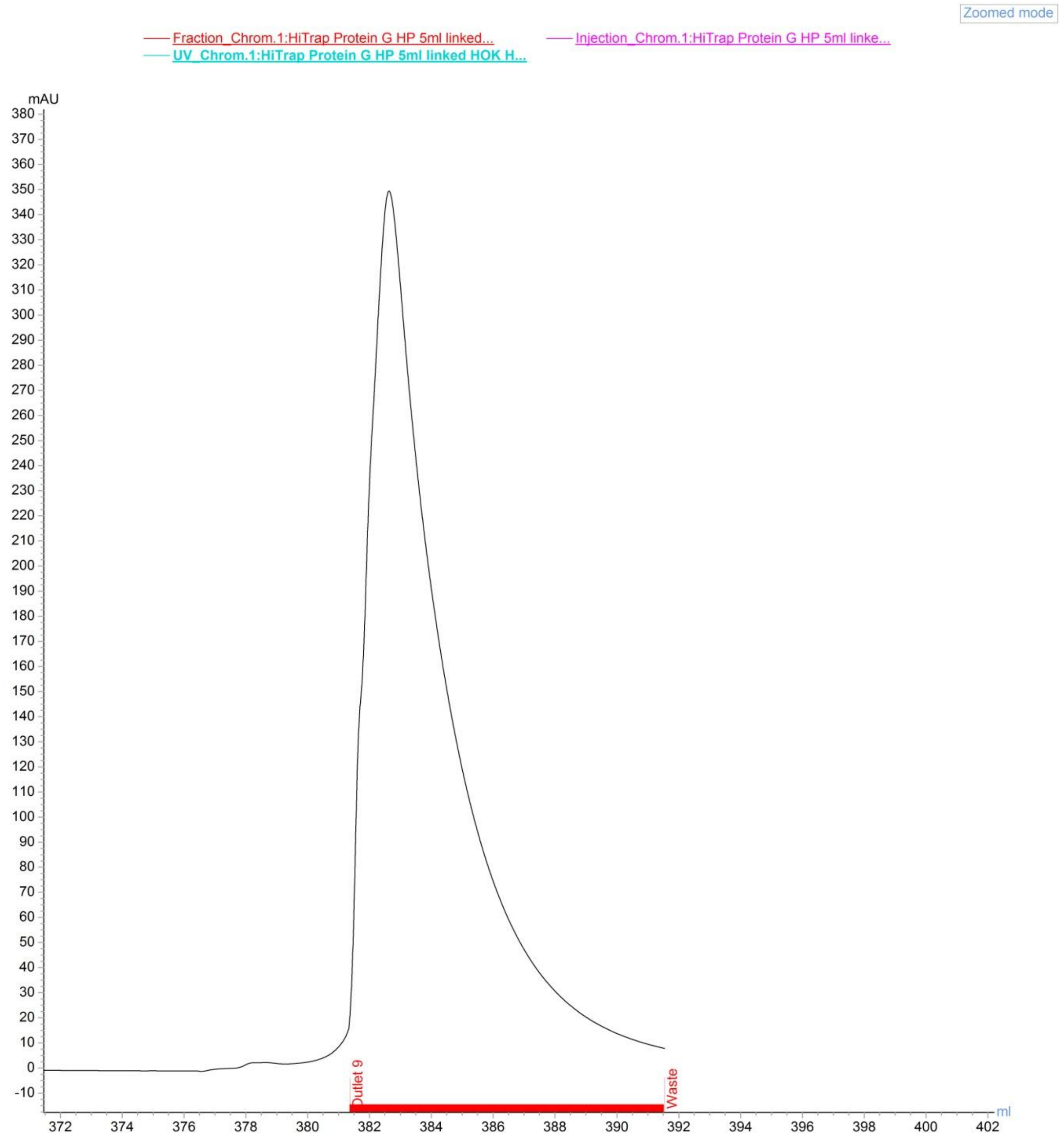

Figure S9f: Trastuzumab IgG1

Figure S9f shows the affinity purification chromatogram profiles of IgG1 using Protein G column. Only the fractions corresponding to mAU >20 were collected as indicated by the bold red line on the X-axis.

X-Axis: ml time scale. Y-axis: mAU absorption as determined by UV detection.

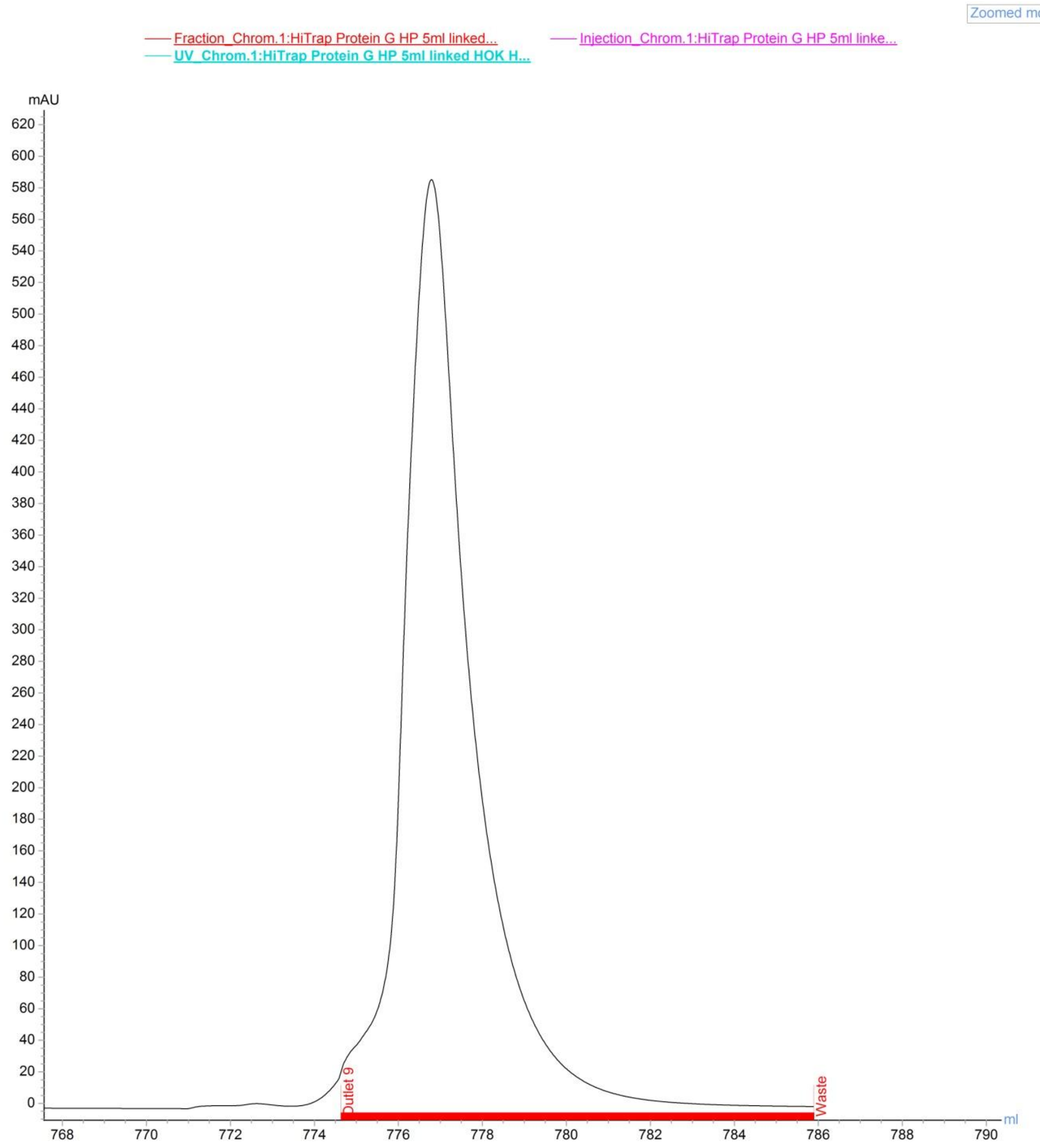

Figure S9g: Trastuzumab IgG2

Figure S9g shows the affinity purification chromatogram profiles of IgG2 using Protein G column. Only the fractions corresponding to mAU >20 were collected as indicated by the bold red line on the X-axis.

X-Axis: ml time scale. Y-axis: mAU absorption as determined by UV detection.

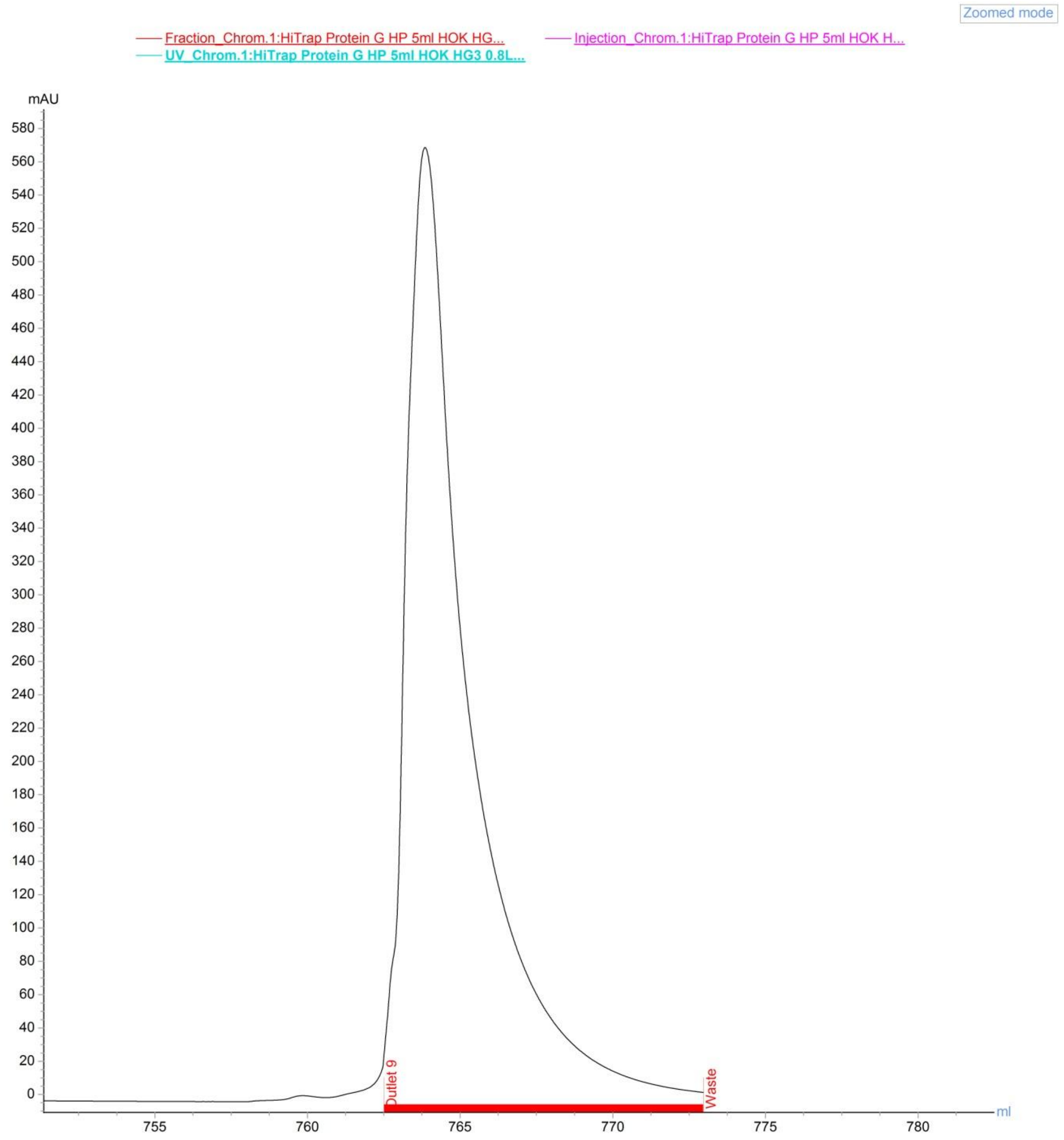

Figure S9h: Trastuzumab IgG3

Figure S9h shows the affinity purification chromatogram profiles of IgG3 using Protein G column. Only the fractions corresponding to mAU >20 were collected as indicated by the bold red line on the X-axis.

X-Axis: ml time scale. Y-axis: mAU absorption as determined by UV detection.

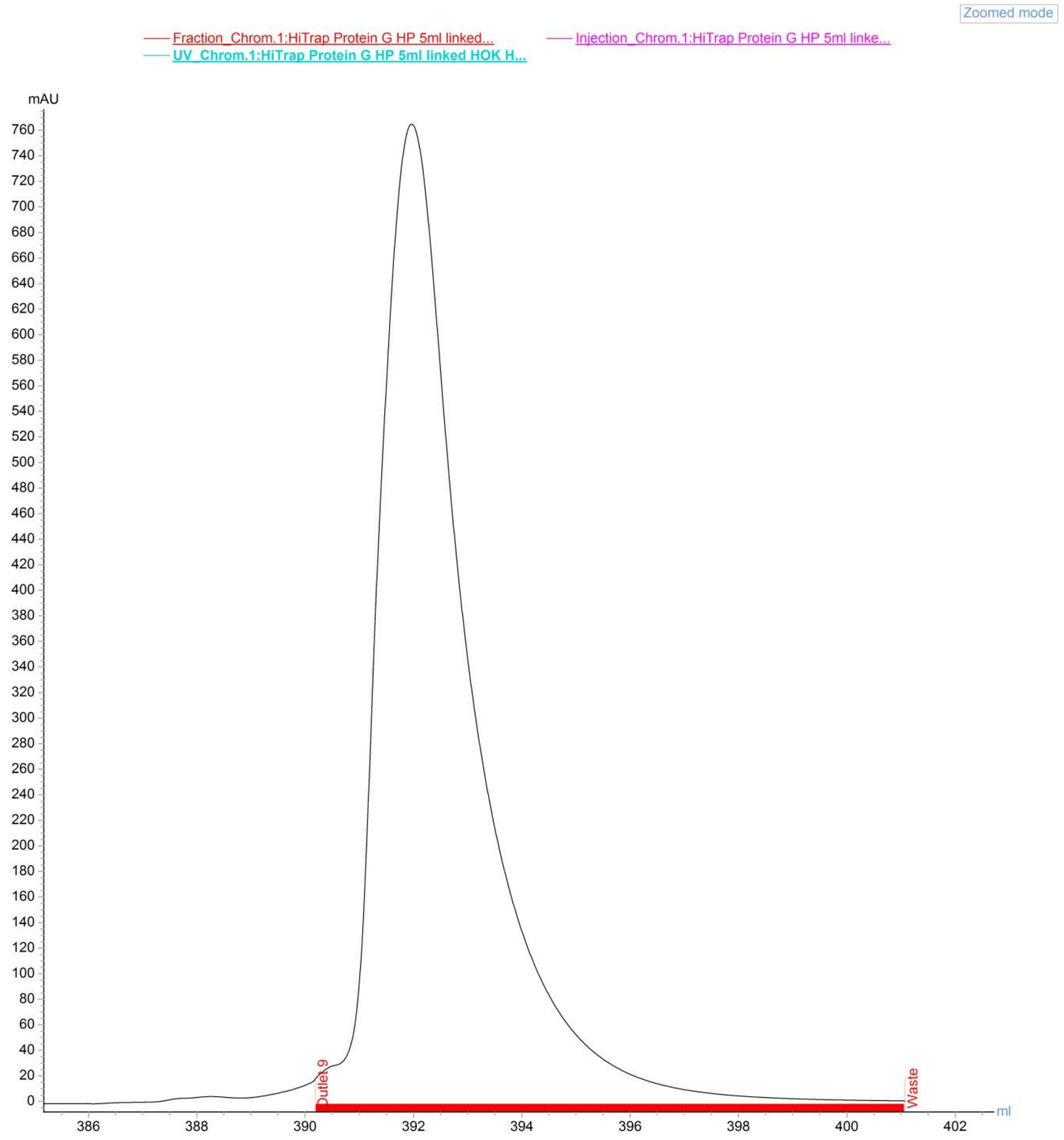

Figure S9i: Trastuzumab IgG4

Figure S9i shows the affinity purification chromatogram profiles of IgG4 using Protein G column. Only the fractions corresponding to mAU >20 were collected as indicated by the bold red line on the X-axis.

X-Axis: ml time scale. Y-axis: mAU absorption as determined by UV detection.

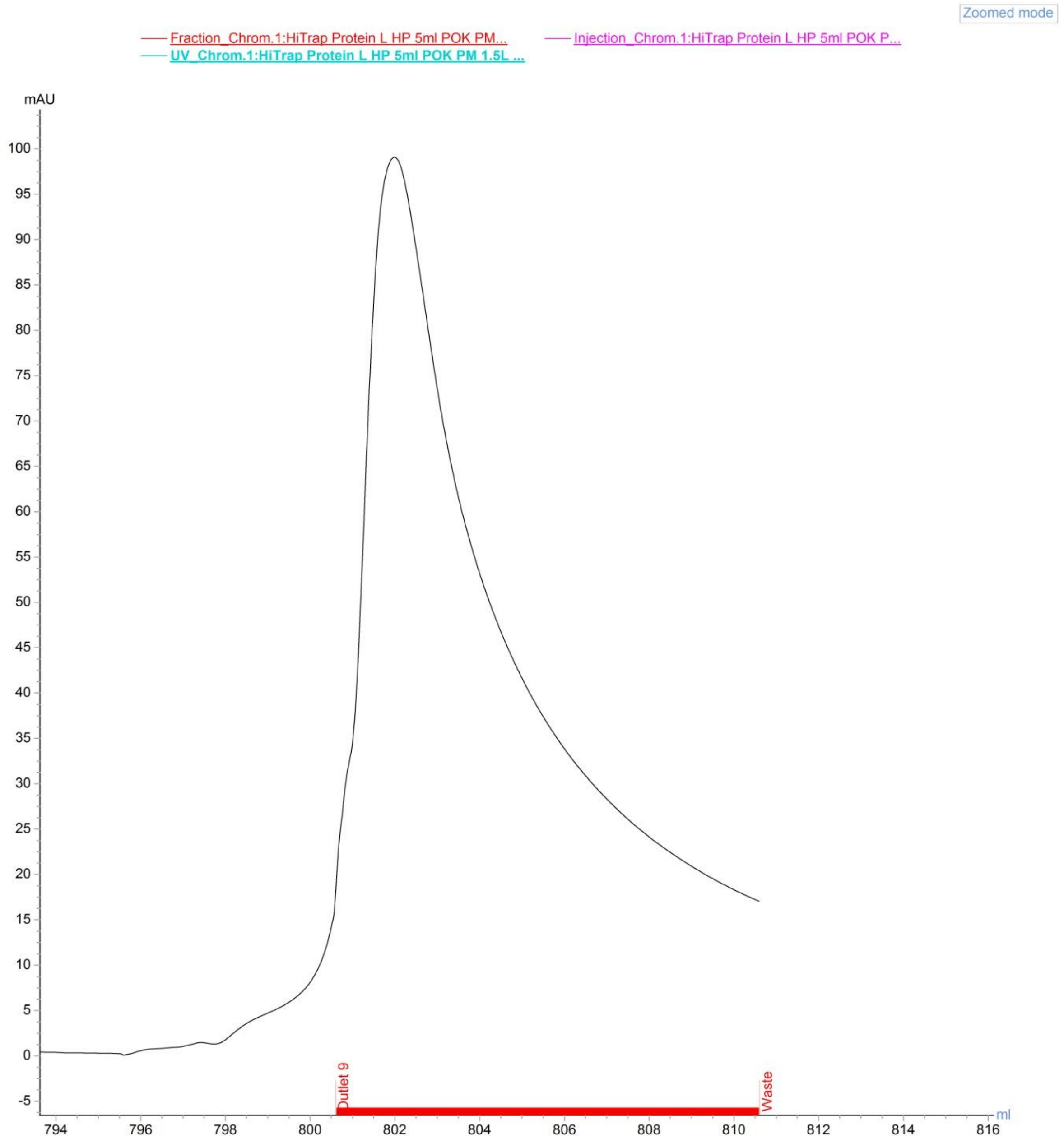

Figure S9j: Pertuzumab IgM

Figure S9j shows the affinity purification chromatogram profiles of IgM using Protein L column. Only the fractions corresponding to mAU >20 were collected as indicated by the bold red line on the X-axis.

X-Axis: ml time scale. Y-axis: mAU absorption as determined by UV detection.

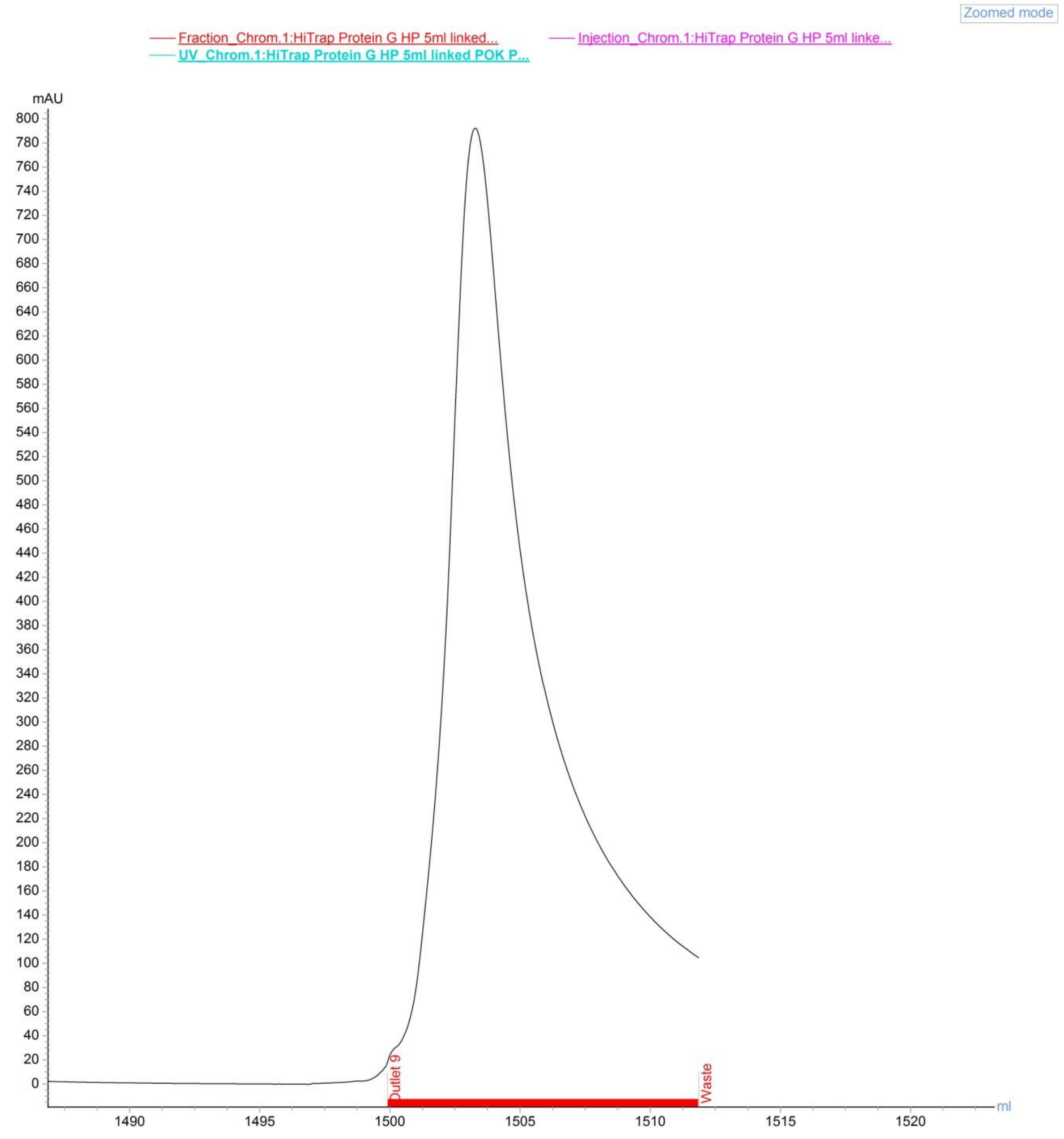

Figure S9k: Pertuzumab IgA1

Figure S9k shows the affinity purification chromatogram profiles of IgA1v using Protein L column. Only the fractions corresponding to mAU >20 were collected as indicated by the bold red line on the X-axis.

X-Axis: ml time scale. Y-axis: mAU absorption as determined by UV detection.

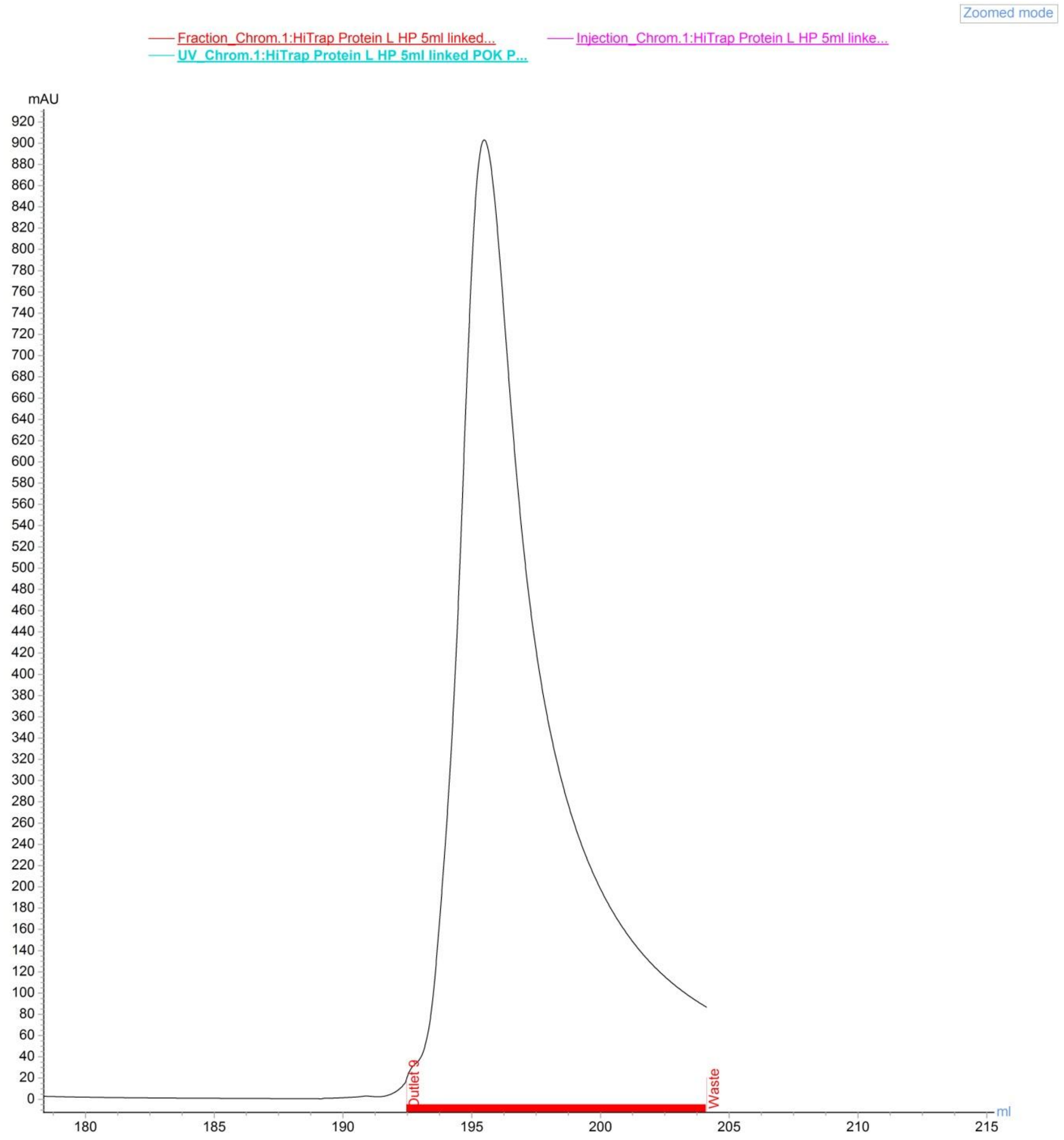

Figure S9l: Pertuzumab IgA2

Figure S9l shows the affinity purification chromatogram profiles of IgA2 using Protein L column. Only the fractions corresponding to mAU >20 were collected as indicated by the bold red line on the X-axis.

X-Axis: ml time scale. Y-axis: mAU absorption as determined by UV detection.

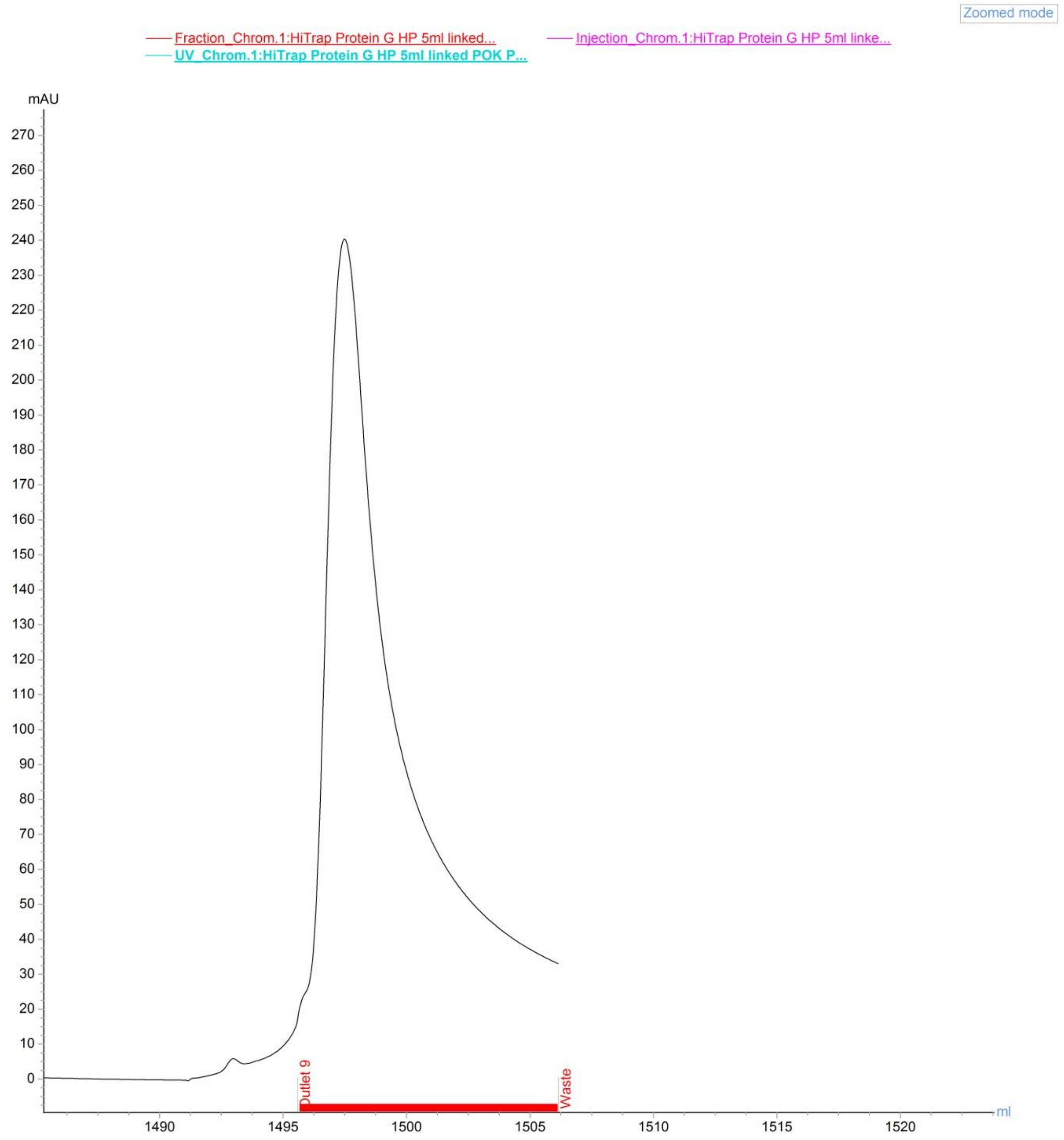

Figure S9m: Pertuzumab IgD

Figure S9m shows the affinity purification chromatogram profiles of IgD using Protein L column. Only the fractions corresponding to mAU >20 were collected as indicated by the bold red line on the X-axis.

X-Axis: ml time scale. Y-axis: mAU absorption as determined by UV detection.

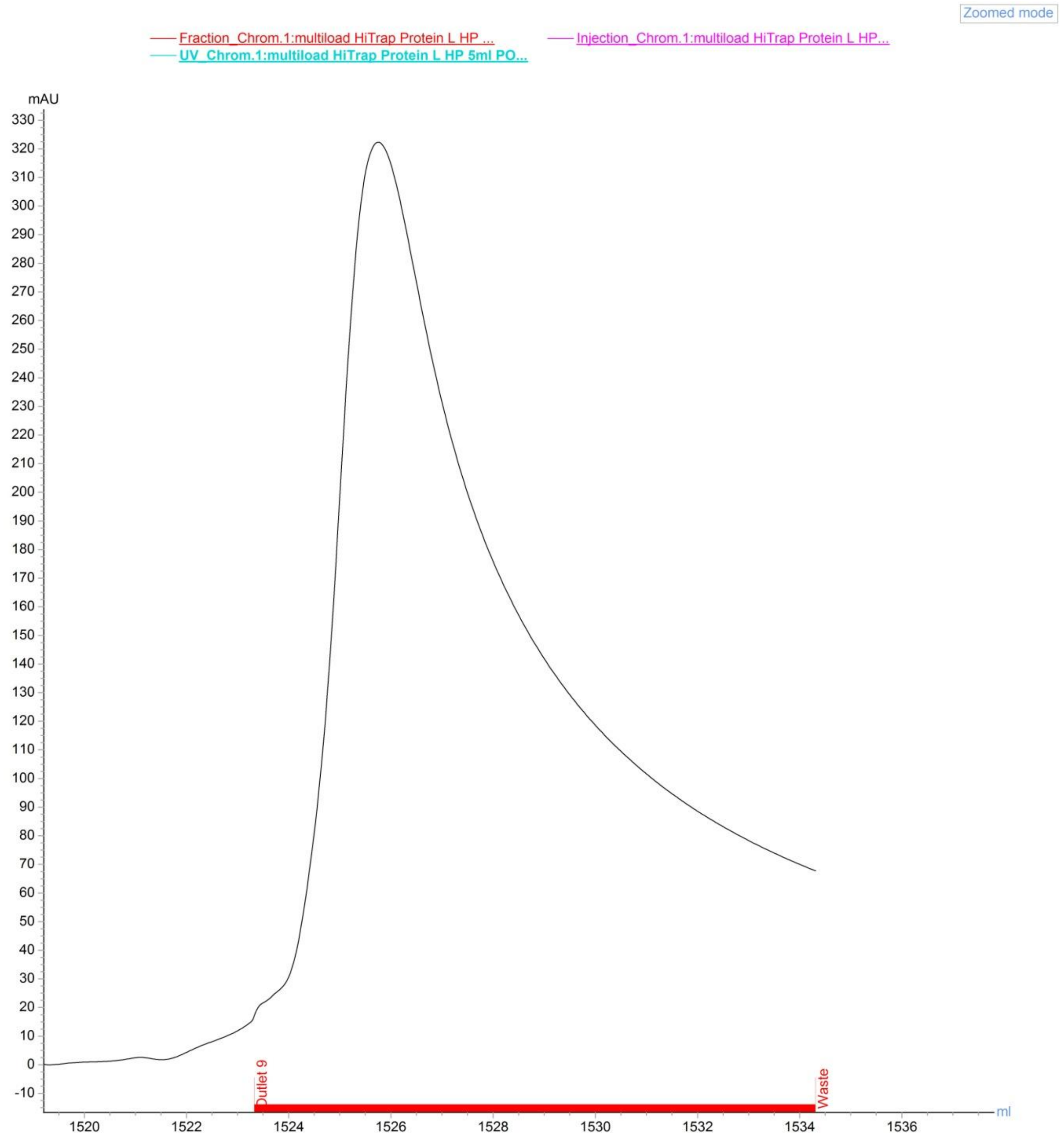

Figure S9n: Pertuzumab IgE

Figure S9n shows the affinity purification chromatogram profiles of IgE using Protein L column. Only the fractions corresponding to mAU >20 were collected as indicated by the bold red line on the X-axis.

X-Axis: ml time scale. Y-axis: mAU absorption as determined by UV detection.

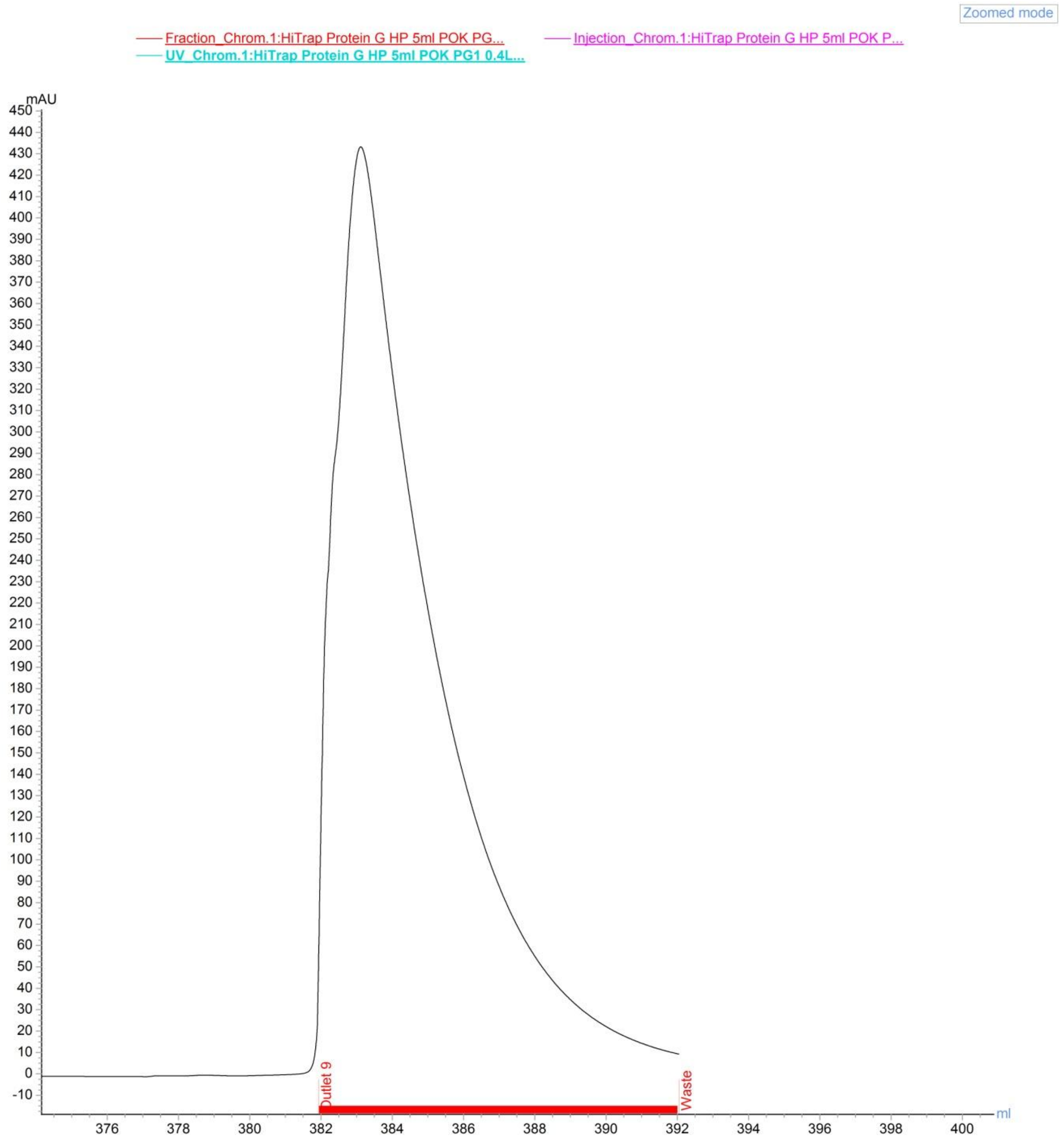

Figure S9o: Pertuzumab IgG1

Figure S9o shows the affinity purification chromatogram profiles of IgG1 using Protein G column. Only the fractions corresponding to mAU >20 were collected as indicated by the bold red line on the X-axis.

X-Axis: ml time scale. Y-axis: mAU absorption as determined by UV detection.

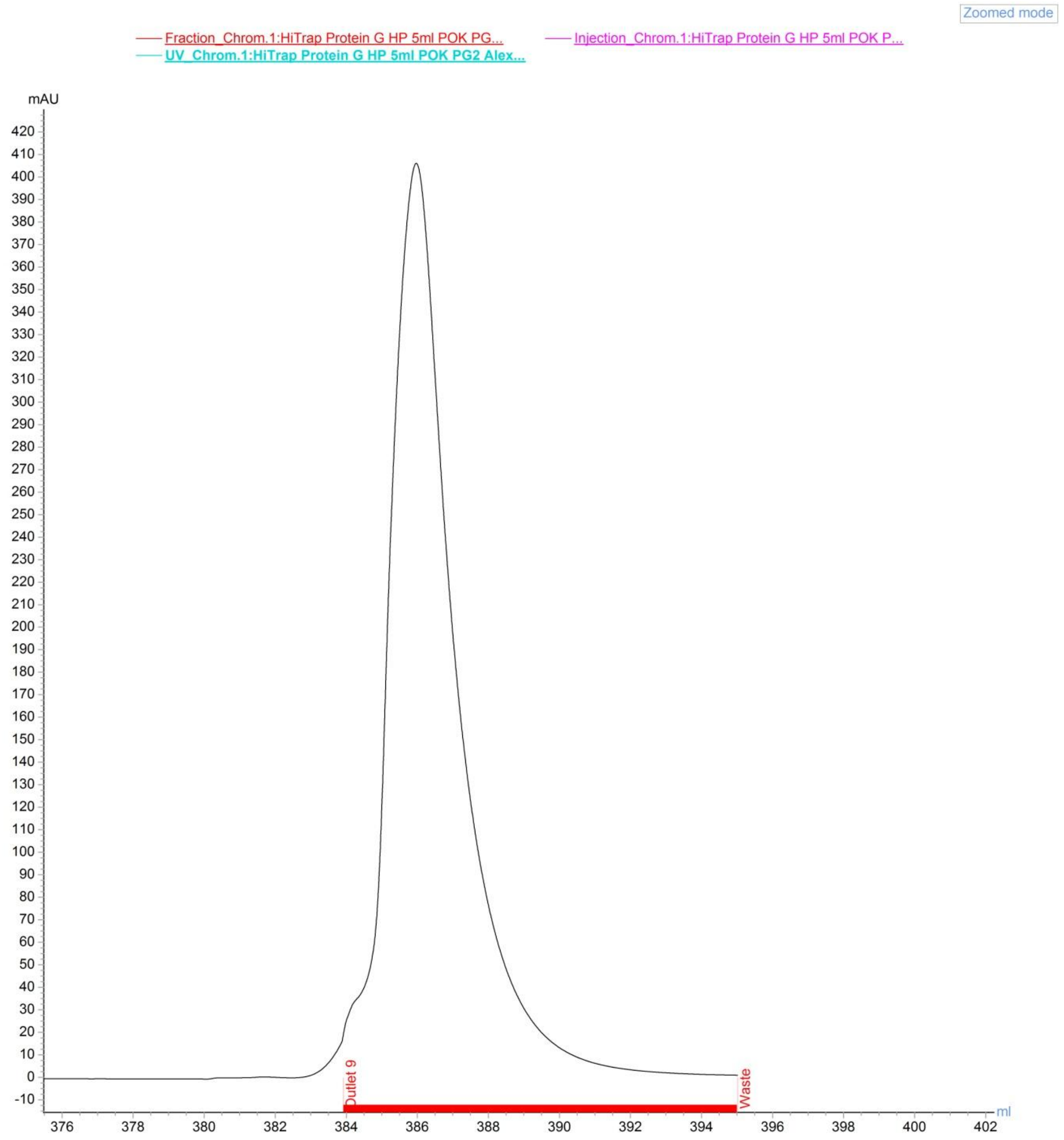

Figure S9p: Pertuzumab IgG2

Figure S9p shows the affinity purification chromatogram profiles of IgG2 using Protein G column. Only the fractions corresponding to mAU >20 were collected as indicated by the bold red line on the X-axis.

X-Axis: ml time scale. Y-axis: mAU absorption as determined by UV detection.

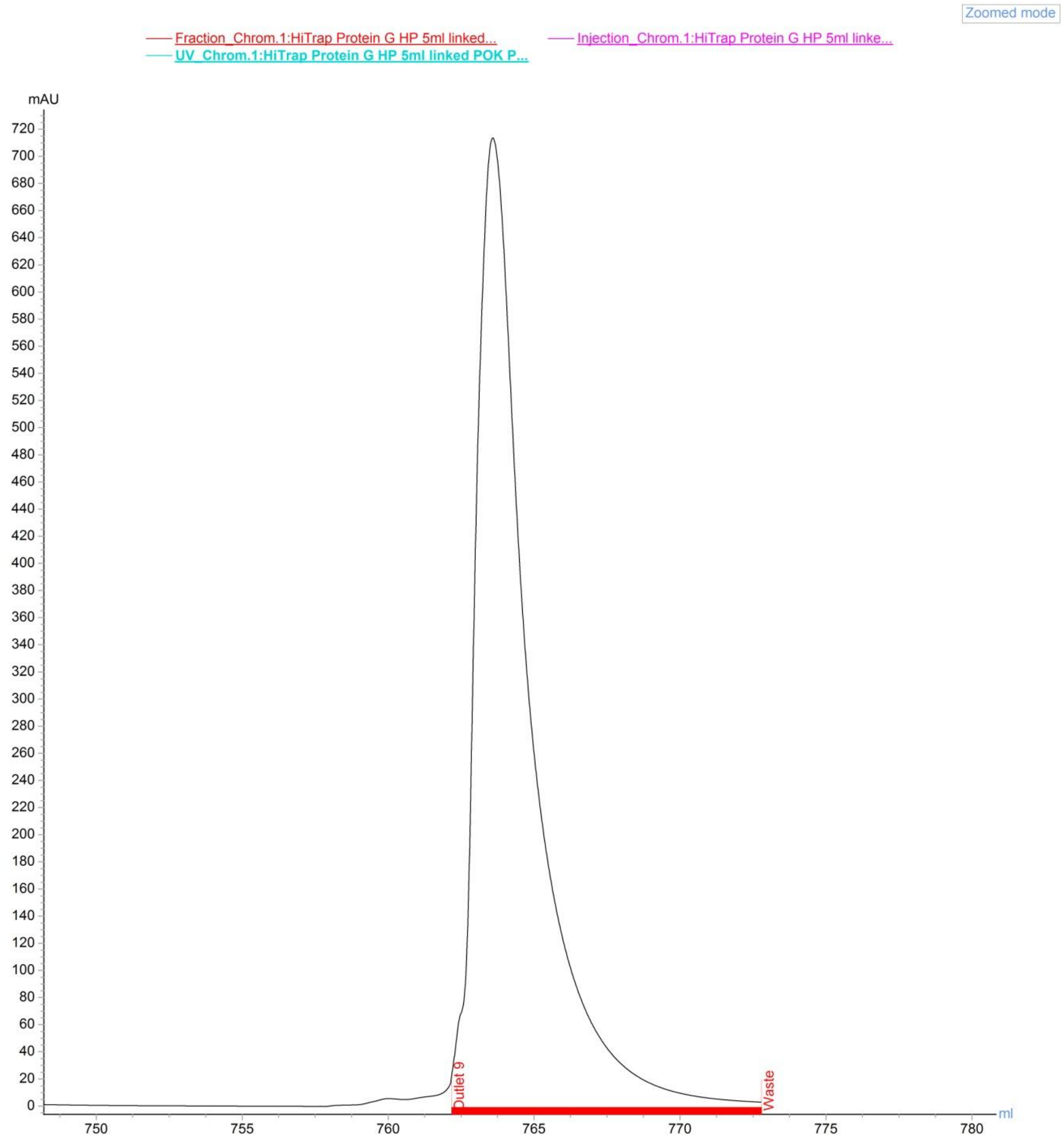

Figure S9q: Pertuzumab IgG3

Figure S9q shows the affinity purification chromatogram profiles of IgG3 using Protein G column. Only the fractions corresponding to mAU >20 were collected as indicated by the bold red line on the X-axis.

X-Axis: ml time scale. Y-axis: mAU absorption as determined by UV detection.

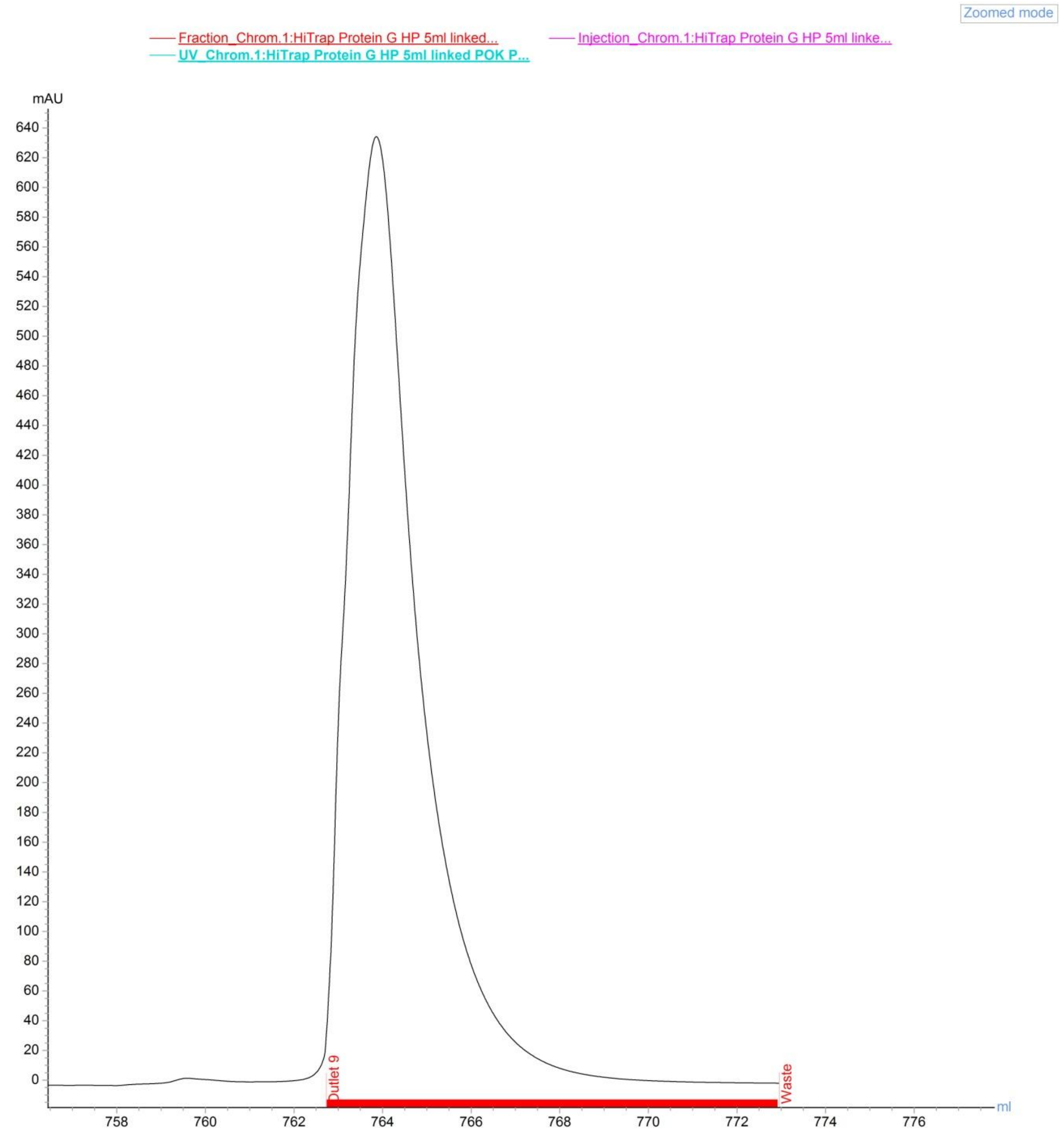

Figure S9r: Pertuzumab IgG4

Figure S9r shows the affinity purification chromatogram profiles of IgG4 using Protein G column. Only the fractions corresponding to mAU >20 were collected as indicated by the bold red line on the X-axis.

X-Axis: ml time scale. Y-axis: mAU absorption as determined by UV detection.

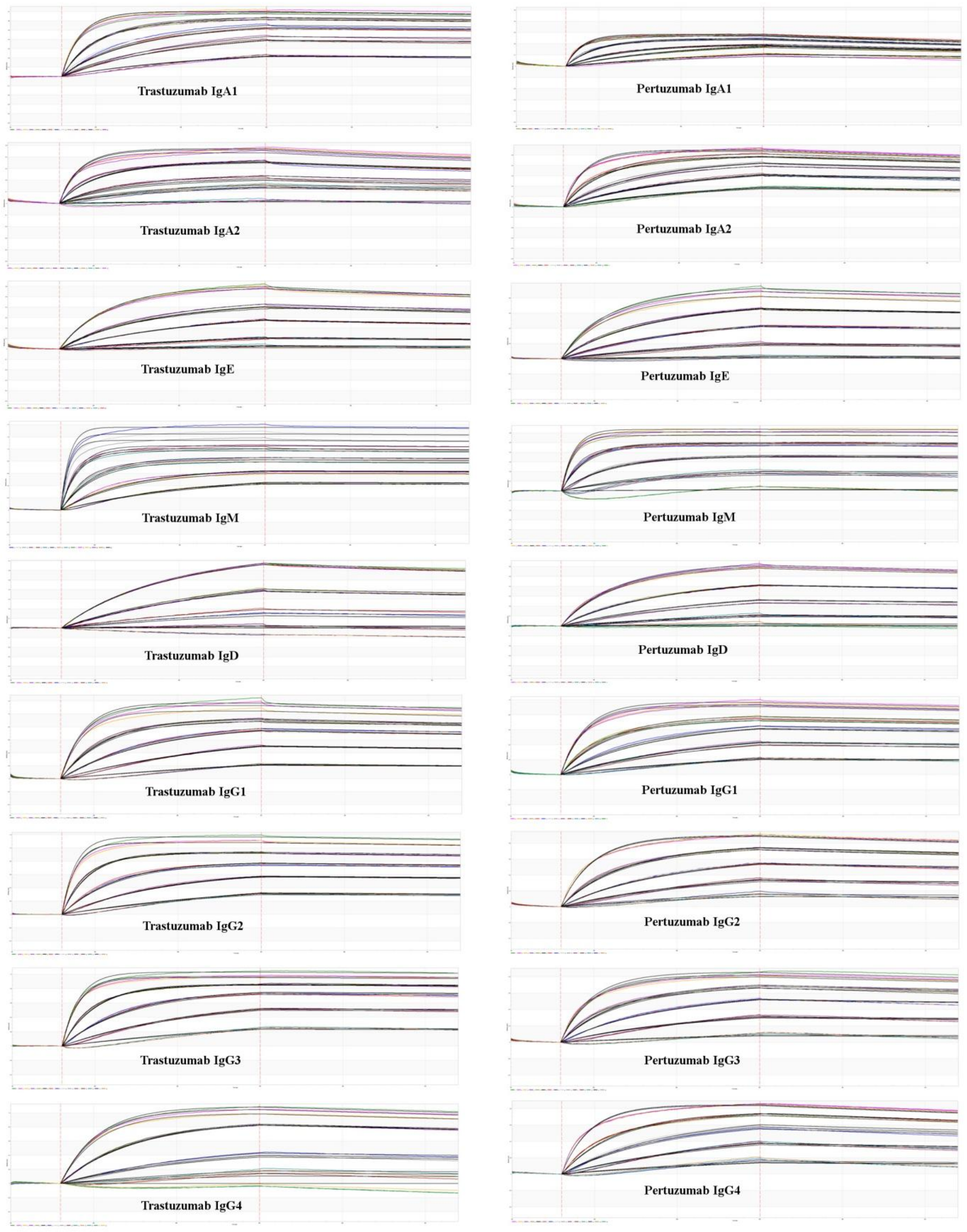

Figure S10a: Binding kinetics of Trastuzumab and Pertuzumab isotype variants using Blitz (with fitting).

The graphs showing the binding kinetics of Trastuzumab and Pertuzumab to Her2 (loaded on NTA sensor) using Blitz (ForteBio). The fitting (black line overlay) is as calculated by the Blitz software.

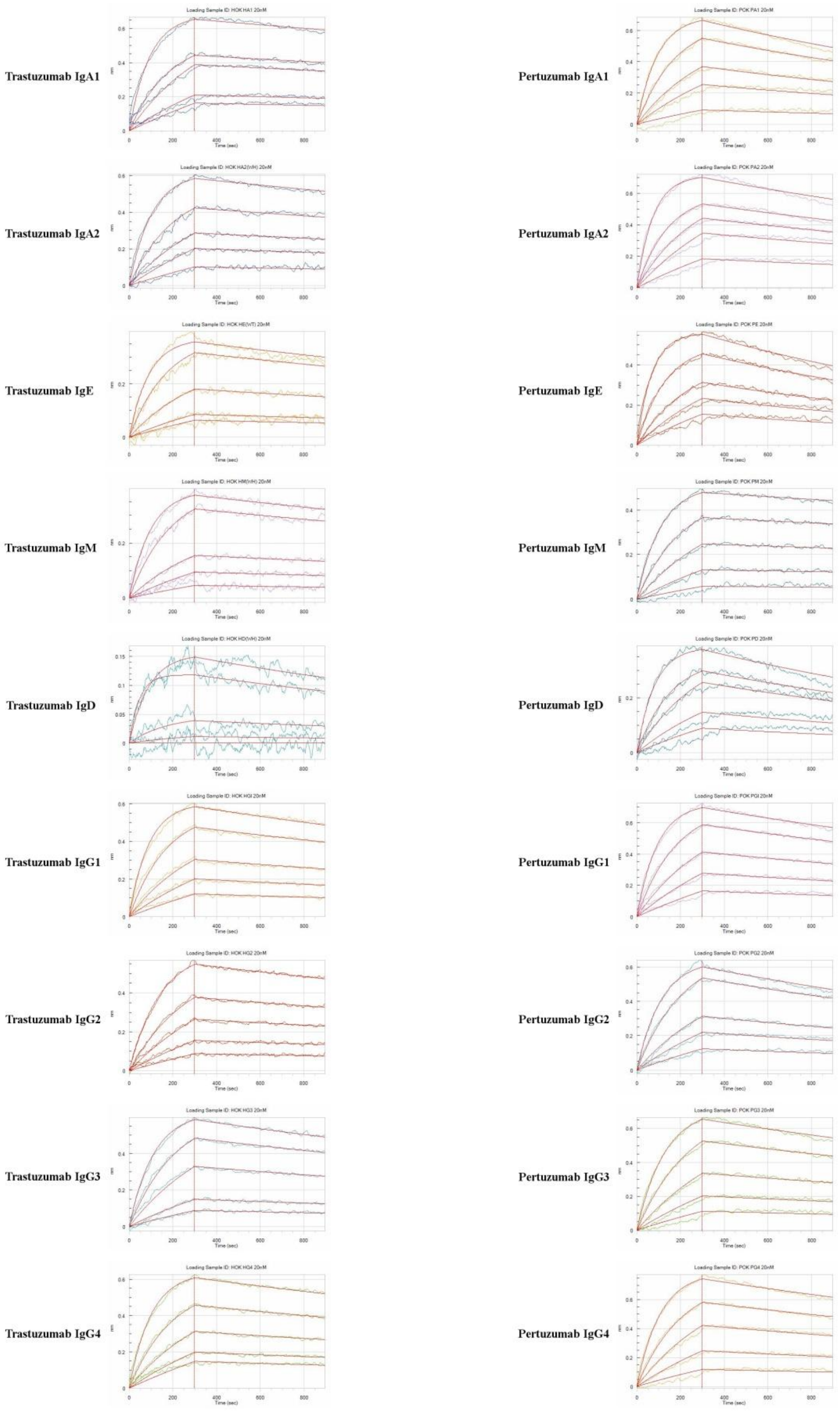

Figure S10b: Binding measurements of Trastuzumab and Pertuzumab isotype variant using Octet (with fitting).

Graphs showing the binding kinetics of Her2 to Trastuzumab or Pertuzumab isotype variants (loaded on ProL sensor) using Octet (ForteBio). Fitting (red line overlay) is as calculated using the Octet software.
